# Linking deliveries to newborns using nationwide Medicaid data

**DOI:** 10.1186/s12874-025-02688-x

**Published:** 2025-10-24

**Authors:** Lilla Orr, Basil Seif, Sun Jeon, Elisa Cascardi, Sakshina Bhatt, Jonas Swartz, Maria Isabel Rodriguez, Lee Sanders, Fernando Mendoza, Jens Hainmueller

**Affiliations:** 1 University of Richmond, Richmond, USA; 2 Stanford University, Stanford, USA; 3 Duke University, Durham, USA; 4 Oregon Health and Science University, Portland, USA

**Keywords:** Maternal and child health, Medicaid, Record linking, Deterministic algorithm

## Abstract

**Background:**

Linking mothers to their newborns in health records is crucial for understanding the impact of policies, programs, and medical treatments on intergenerational health outcomes. While previous studies have used shared identifiers for linkage, such data are often unavailable in Medicaid records due to privacy concerns. Existing algorithms are not sufficiently flexible to accommodate Medicaid data from all states and from both Medicaid Analytic Extract (MAX) and Transformed Analytical Files (TAF) data systems.

**Methods:**

We present a scalable framework and linking algorithm that connects deliveries and infants without relying on names, addresses, or linkage to vital records. First, we confirm our ability to identify newborn beneficiaries and deliveries resulting in live birth across states and over time by comparing our findings to the total number of Medicaid births recorded in the National Vital Statistics System (NVSS). Second, we confirm that our algorithm accommodates variations in Medicaid records over time and across states for MAX and TAF data, supporting matches at different levels of stringency. Finally, we assess the extent to which our algorithm is effective across demographic groups.

**Results:**

Using data from all 50 states over 9 years, our algorithm linked 11.68 million mother-infant dyads, covering 68% of Medicaid-enrolled infants, over 30% of all U.S. infants. Our linked cohort is approximately representative of the broader population of Medicaid beneficiaries on key observable characteristics including race and ethnicity, age, gender, and region. However, linked beneficiaries are more likely to be white and from the Midwest or Northeast relative to those we are unable to link.

**Conclusions:**

Despite substantial variation in the nature of Medicaid data across states and over time, it is possible to identify family units in all states between 2011 and 2019 without linking claims to vital records. This algorithm will facilitate research on social determinants of health and the intergenerational effects of medical interventions and public policy.

**Supplementary Information:**

The online version contains supplementary material available at 10.1186/s12874-025-02688-x.

## Introduction

Understanding the health of infants and young children requires considering family contexts. However, to protect privacy, health records often lack linkages or shared identifiers, such as names or addresses, between parents and children, making it challenging to study family and intergenerational health effects. For births covered by Medicaid, we introduce a method to link infants and mothers^1^ without relying on any information that would facilitate linkage to vital records.

Medicaid has covered approximately 42% of U.S. births in recent decades [1, 2]. Accurately establishing these connections while maintaining privacy is crucial for research on social determinants of early childhood health [3, 4], the impact of medication use during pregnancy on child health [5–7], and the intergenerational effects of public policy [8, 9]. Even summaries of health outcomes for mothers and young medicaid beneficiaries may be incomplete without the ability to observe both beneficiaries’ claims for care related to the delivery [4].

Given that Medicaid funds nearly half of all births nationwide, analyzing Medicaid beneficiaries provides valuable insights into the health of people giving birth and young children. Although this population is not representative of the entire nation, studying outcomes among Medicaid beneficiaries is critical when evaluating policies aimed at reducing health disparities and for understanding potential unintended consequences of public policy for low-income Americans.

Building on earlier research [10–16], we first aim to develop a detailed framework for identifying newborns and deliveries that accounts for variations in Medicaid records over time and across states. Addressing significant discrepancies in how states document Medicaid spending is essential for developing an algorithm that can be applied nationwide. Past attempts to identify and link deliveries and newborns within Medicaid have typically focused on one state or a small number of states [8, 10, 13, 17]. Additionally, covering the transition from the MAX to the TAF Analytic Files as well as the ICD-9 to ICD-10 transition is essential for the analysis of timeseries data that includes the 2010s. In addition to excluding several states, past efforts to link Medicaid deliveries and newborns nationwide have relied exclusively on MAX data [5, 18, 19]. Because TAF significantly expands the availability of Medicaid data to researchers, the development of a method to identify infant-mother dyads in TAF data has been identified as a priority for the study of U.S. maternal health [20].

Second, we aim to validate our procedures for identifying newborns and deliveries in Medicaid claims by comparing the number of identified newborns and deliveries to expected counts based on micro-data from the NVSS. Past research has identified significant deviations between the total number of births expected to appear in Medicaid claims and the number identified by state, so we aim to assess the efficacy of our procedure across states [21, 22].

Third, we aim to implement a deterministic linking algorithm that does not require Medicaid claims to be linked to vital records or other beneficiary-level data, as is often needed for identifying family units within Medicaid [13, 23, 24]. We aim to avoid any reliance on electronic health records, private insurance data, or other non-governmental sources [25–28], thereby protecting the privacy of beneficiaries. We additionally aim to include groups often excluded from infant-mother linkages, such as those without full Medicaid coverage^2^ or those enrolled for only short periods [18]. Excluding these groups can limit the scope of analyses focused on social determinants of health.

Overall, we aim to develop a procedure that yields high identification rates across various demographic groups. Our algorithm is designed to be as inclusive as possible, while also being adaptable for researchers with different needs.^3^ By identifying and linking newborns to deliveries in all states, while bridging MAX and TAF, we aim to enable large-scale longitudinal analyses of maternal and child health in the United States.

## Data and methods

### Data

Our analysis draws on data from MAX from 2011 to 2015 and TAF spanning from 2014 to 2019. Our MAX data analysis utilizes Personal Summary, Inpatient, and Other Services files. Similarly, our TAF data analysis utilize Demographic Eligibility, Inpatient and Other Services files. All data were acquired directly from Centers for Medicaid and Medicare Services (CMS). Details regarding data acquisition can be found in Appendix 1.

We do not rely on any external information regarding beneficiaries. We use information from providers to determine facility ZIP codes. For physicians and physician groups providing care for deliveries and newborns, we link the National Provider Identifier (NPI) appearing in MAX and TAF claims to records from the National Plan and Provider Enumeration System (NPPES). Specifically, we utilize the ZIP code of the employment address that was registered for each NPI during the year in which the claim was filed. For further details on our processing of NPPES data, see Data Acquisition Appendix 1.

Our methodology for linking deliveries and infants involves three steps. First, we identify deliveries of living singleton newborns. Second, we identify singleton newborns. Third, we link the two groups using our linking algorithm.

### Identification of deliveries

To identify deliveries, we leverage a comprehensive list of International Classification of Diseases (ICD-9 and ICD-10) and Current Procedural Terminology (CPT) codes to capture claims indicating deliveries resulting in live births. Our list includes 96 distinct codes, detailed in Appendix 2, which are derived from Auty et al. [16, 29, 30] method 4 and Sarayani et al. [14]. To avoid false positives, we excluded codes that may suggest delivery but are also commonly used in claims for antepartum or postpartum care, or are frequently used when deliveries do not result in live birth. Further information on our process for refining this code list is available in Appendix 2.

Using this code list, we identified all claims^4^ that list these codes across all inpatient and other services files. We then identified the beneficiary of each claim and retained those who were between the ages of 12 and 50 at the time of the claim.

Next, we mapped the delivery claims for each beneficiary to unique, distinct deliveries, as a single delivery may generate multiple claims on different days, and some beneficiaries may have had multiple deliveries over our nine-year study period. We used a hierarchical clustering algorithm to group the delivery claims into unique delivery clusters. Clustering was based on dates, with all claims within a given time period assumed to be associated with the same delivery. We required that the midpoint of each delivery period be at least 270 days apart from the midpoint of any other delivery period to ensure that data errors would not lead us to incorrectly assume that a beneficiary had two deliveries resulting in live births within a nine-month span.^5^

Finally, we validated our procedure by comparing the number of deliveries we identified (i.e., the number of delivery claim clusters) to the number of deliveries recorded in the CDC’s National Vital Statistics System (NVSS) for which Medicaid is listed as a payer. We conducted this comparison at the state-year level to account for variations in Medicaid claims reporting, diagnostic and procedure code usage, and data processing systems at the state and federal levels over time and space.

### Identification of newborns

To identify newborns, we utilized ICD-9, ICD-10, and CPT codes that indicate care for newborns. We compiled these codes by integrating lists from Research Data Assistance Center (ResDAC) resources for identifying newborn care^6^ and conducting an original review of annual medical coding manuals. The complete list of codes is provided in Appendix 3. We identified all claims containing these codes billed for beneficiaries from their birth date up to 7 days post-birth.

However, this method alone is insufficient to identify newborns nationwide. In several states, care for newborns is often billed under their mother’s coverage, making it impossible to directly observe the identity of the newborn receiving care based solely on claims data [31].

To address this limitation, we employed an additional method that does not rely on diagnosis and procedure codes. Specifically, we identified state-years where the number of newborns detected using our list of diagnosis and procedure codes was less than 95% of the total number of newborns recorded in the NVSS records. For these state-years (listed in Appendix 3), we included all beneficiaries who were enrolled in Medicaid before or up to one day after their date of birth in our universe of newborns, utilizing enrollment dates from the enrollment files. The rationale is that newborns enrolled in Medicaid at or near the time of birth should be considered as candidates for matching because they are likely born to beneficiaries who are also included in the Medicaid claims.

### Matching algorithm

To link mothers to newborns, we use a greedy deterministic matching algorithm, where each unmatched infant can be linked to only one unmatched delivery and vice versa. The matching is based on the state of residence, newborn’s date of birth, delivery date window, and four demographic variables: (1) residential ZIP code of the beneficiary (mother or newborn), (2) ZIP code of the facility where the delivery or newborn care occurred, (3) race/ethnicity of the beneficiary, and (4) beneficiary’s case number. The underlying premise is that infants must be born at approximately the same time and place as the delivery occurs, and the beneficiaries should share attributes that we expect mothers and infants to have in common (such as ZIP codes).

We exclude newborns for which we cannot confidently determine the birth date using enrollment records and exclude deliveries for which we cannot identify a narrow range of plausible dates for the delivery window using associated claims. We identify the residential ZIP code, case ID number, and racial or ethnic identity of each beneficiary based on enrollment data. We use NPIs to estimate facility ZIP codes. We remove invalid ZIP codes and case ID numbers assigned to more than 10 beneficiaries in a given state-year, as we do not expect these to have been assigned at the household level. We also remove indicators of multi-racial identity, as we anticipate relatively low congruence between the racial and ethnic identity of infants and mothers (as recorded in Medicaid enrollment data) when either is multiracial. Data processing details and a summary of data missingness across matching variables are outlined in Appendix 4.

The matching algorithm proceeds in six phases, each involving progressively less restrictive criteria. Across all phases, we always require that the state of the newborn matches the state of the mother and that the newborn’s date of birth falls within a narrow range around the delivery date window. Matches are accepted only if there is a unique delivery-newborn pair that satisfies the criteria. This helps prevent incorrect matches. Any linked newborns and deliveries are removed from the unmatched pool, and the algorithm proceeds to identify unique matches using slightly less stringent criteria. A detailed summary of the criteria for each matching phase is provided in Appendix 5.

In phase one, we impose the most stringent criteria. We require that the newborn’s date of birth falls within the estimated delivery date range and that the newborn and mother share the same unique case number. A shared case ID is a strong indicator of a shared household [12]. Additionally, we require that they share the same facility ZIP code, residential ZIP code, and racial or ethnic identity. For these three variables, we iterate through eight steps, requiring that newborns and mothers match on all three, any two, any one, or none of these variables (see Appendix 5 for details).

In phases two and three, we repeat the steps from phase one but allow for a one- or two-day deviation from the estimated delivery date range. In phases four through six, we relax the criteria further by allowing for missing or conflicting case IDs. This is necessary because some states do not use case ID numbers, and others may assign different numbers to parents and their children. We begin by looking for unique matches for infants born within the estimated delivery date range, with shared residential and facility ZIP codes and race or ethnicity (phase four). Then, the allowable date range is expanded by one day (phase five) and two days (phase six), as in the first three phases.

The sequential, deterministic nature of this algorithm ensures high transparency. Researchers can exclude dyads from specific matching phases from their analyses if they believe the matches were made using insufficiently stringent criteria.

## Results

### Identification: deliveries and newborns

**Fig. 1:**
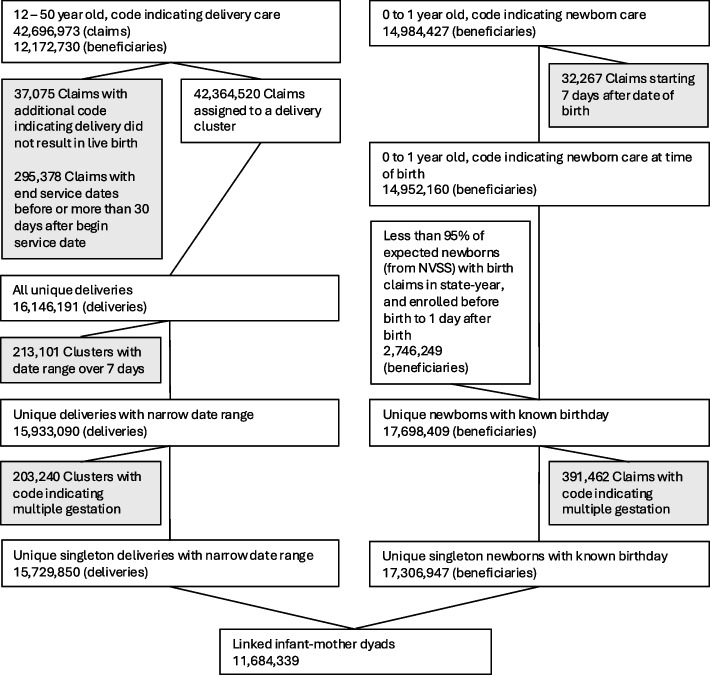
Total Number of Deliveries and Newborns Included in the Analysis. Note: Total sample size at each stage of data processing. Shaded boxes indicate exclusions from further analysis

The aggregated results from our identification of deliveries and newborns, as well as the linking process between mothers and newborns, are presented in Fig. 1.

Across nine years of nationwide Medicaid records, our delivery identification method yields 12,172,730 beneficiaries aged 12 to 50 years with claims indicating they received care for a delivery resulting in a live birth. After consolidating these delivery-related claims into unique deliveries using our hierarchical clustering algorithm, we identify a total of 16,146,191 unique deliveries. A detailed description of the delivery claim clusters can be found in Appendix 2. For the subsequent matching, we exclude 213,101 deliveries (1.32%) for which we are unable to estimate a delivery time window of 7 or fewer days, and 203,240 deliveries (1.26%) that appear to have resulted in multiple live births, based on diagnosis and procedure codes listed in Appendix 3, as our focus is on singleton births.^7^

Using the same universe of Medicaid claims, our newborn identification method identifies 17,698,409 beneficiaries who were covered by Medicaid at the time of birth. This includes 14,952,160 newborns with a birth-related claim under their own beneficiary ID, as well as 2,746,249 beneficiaries from states and years where care for newborns was likely not billed directly to them. In these cases, we identified the newborns based on their Medicaid enrollment at the time of birth. For the subsequent matching, we additionally exclude 391,462 newborns (2.21%) who do not appear to be singletons, based on diagnosis and procedure codes listed in Appendix 3.

While the number of newborns roughly matches the number of deliveries, there are slightly more newborns identified. This discrepancy is likely due to cases where newborns are Medicaid beneficiaries at birth, but their mothers did not use Medicaid insurance for their deliveries. These newborns will later be excluded during the matching process because they cannot be linked to a corresponding delivery.

Next, we compare the number of identified deliveries and newborns against the number of deliveries and newborns covered by Medicaid as reported in the NVSS data. Figure 1 and Table 1 show this comparison at the state level. Note that this comparison is based on the numbers before excluding multiple gestation newborns and deliveries, as these cannot be separately identified in the NVSS data.

**Fig. 2:**
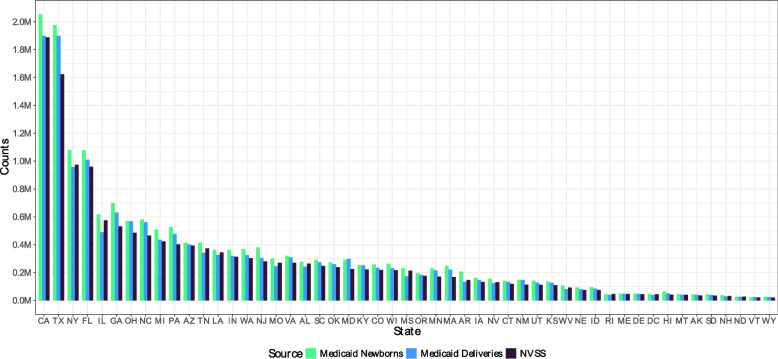
Total Number of Deliveries and Newborns by State. Note: Total number of deliveries and newborns identified prior to removing multiple gestation births, compared to the expected number of deliveries (NVSS). States are listed in order based on the expected number of deliveries

**Table 1:** Total number of deliveries and newborns by state

State	Medicaid newborns	Medicaid deliveries	NVSS
AK	42,725	40,408	35,586
AL	276,782	241,612	264,127
AR	207,117	133,210	146,535
AZ	413,557	401,526	393,541
CA	2,052,060	1,898,824	1,887,956
CO	258,249	234,391	217,812
CT	140,488	133,362	119,130
DC	45,385	38,737	44,267
DE	48,410	47,395	44,590
FL	1,077,199	1,009,418	960,087
GA	699,029	629,917	530,773
HI	63,974	50,534	41,436
IA	161,669	146,920	132,498
ID	94,863	84,824	74,136
IL	617,499	490,460	575,119
IN	361,494	319,439	313,466
KS	137,096	126,112	109,300
KY	252,824	253,366	222,650
LA	362,185	325,154	344,515
MA	249,039	220,788	168,220
MD	314,782	298,090	224,852
MI	509,047	434,382	423,433
MN	229,156	216,985	171,327
MO	302,150	245,449	270,548
MS	231,286	174,229	214,290
MT	45,360	42,394	40,569
NC	581,232	561,033	464,841
ND	26,273	25,559	26,872
NE	92,780	80,290	74,642
NH	37,369	29,352	31,588
NJ	380,103	304,896	280,903
NM	148,465	145,899	112,806
NV	155,225	124,059	131,066
NY	1,078,669	958,286	973,559
OH	570,030	568,549	485,133
OK	272,423	260,261	238,201
OR	196,409	184,314	176,276
PA	527,308	476,776	401,975
RI	45,049	39,871	46,642
SC	291,027	274,293	247,401
SD	41,081	38,231	34,576
TN	415,537	342,279	373,576
TX	1,976,134	1,898,492	1,623,597
UT	141,615	127,946	111,623
VA	320,422	308,333	270,162
VT	23,049	23,802	21,334
WA	368,707	324,330	303,665
WI	263,242	231,648	217,579
WV	106,512	82,542	91,366
WY	25,553	25,162	20,292

Note: Numeric values plotted in Fig. 2

We find a high degree of congruence between our identification of newborns and deliveries in the Medicaid data and the numbers reported in the NVSS across all states. These patterns are largely consistent across states and over time, as discussed in Appendices 2 and 3. Our identification consistently yields slightly higher numbers of deliveries than those reported in the NVSS. Overall, our identification using Medicaid claims yields 9.28% more deliveries than reported in the NVSS data as being covered by Medicaid. For newborns, our identification yields 19.78% more newborns than reported in the NVSS. This over-coverage is likely due to newborns enrolled in Medicaid at birth whose mothers did not use Medicaid for the delivery, misreporting in the payer variable in the NVSS data, or other discrepancies between the two datasets.^8^ However, this over-coverage is not a significant problem for matching, as newborns not born under Medicaid will go unmatched.

### Linked cohort

**Fig. 3:**
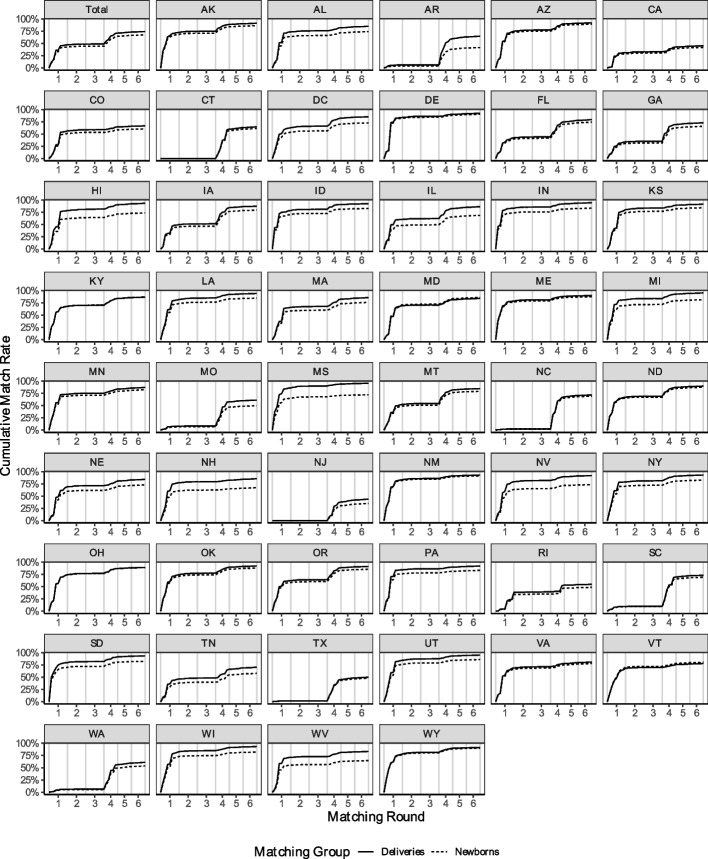
Cumulative Match Rate Nationwide and by State. Note: Each plot shows the cumulative percentage of deliveries (solid lines) and newborns (dotted lines) linked at each step of the matching algorithm. Divergences between the lines indicate instances where there were more newborns than deliveries eligible for matching. The first plot shows nationwide results, while the remaining plots show state-level results

Figure 3 displays the results of the matching algorithm by phase, both for the total cohort (top-left panel) and for each state. Detailed results for every step are provided in Appendix 5. Overall, through all six phases, our matching algorithm results in a total of 11,684,339 mother-newborn dyads. This represents 74.28% of deliveries matched to newborns and 67.51% of newborns matched to a delivery.^9^

Encouragingly, a substantial portion of these matches are based on very strict criteria. In phase one, which requires newborns and mothers to match on delivery time, case numbers, and combinations of residential ZIP code, facility ZIP code, and race/ethnicity, we identify 7,432,402 mother-newborn dyads. This corresponds to 42.94% of newborns and 47.25% of deliveries.

Repeating the steps from phase one but expanding the delivery window by 1 and then 2 days in phases two and three results in only a limited number of additional matches. At the end of phase two, we have identified 7,661,860 mother-newborn dyads, and at the end of phase three, 7,689,211 dyads. Since all these matches are based on exact case number matches, there is reason to be confident in their accuracy.

What is missing from phases one to three are states that do not assign case ID numbers at the household level, including states such as Texas, New Jersey, and Connecticut. To capture matches in these states, phases four to six exclude the case ID requirement, instead matching mothers and newborns based on date, location, and race/ethnicity. As shown in Fig. 3, this adjustment results in a significant number of additional matches. By the end of phase four, there are 11,190,695 linked mother-newborn dyads, representing 71.14% of all deliveries and 64.66% percent of all newborns. This increase is most pronounced in states that do not report case ID numbers in the Medicaid data. At the end of phases five and six, there are 11,525,870 and 11,684,339 mother-newborn dyads, respectively.

Figure 3 illustrates that the algorithm is flexible enough to identify a large number of family units in every state, despite variations in data quality and reporting procedures. For newborns, linking rates range from 35.08% in New Jersey to 91.42% in New Mexico, with a median rate of 79.09%. State-specific linking rates at the end of phases three and six are available in Appendix 5.

To assess the representativeness of the linked cohort compared to the total universe of deliveries and newborns, we examined whether the linked cohort broadly represents all Medicaid beneficiaries on observable dimensions. Table 2 presents the demographics of these beneficiaries before and after linking. For both deliveries and newborns, there is no meaningful difference between the demographics of those we identified and those we attempted to match. The linked cohort is slightly more likely to include beneficiaries from Midwestern and Northeastern states and who are non-Hispanic white or non-Hispanic Black, and slightly less likely to include those from Southern and Western states or those who are Hispanic or have unknown race/ethnicity, relative to those who we are unable to link.^10^ Newborns are also substantially less likely to have race or ethnicity recorded in their enrollment records relative to adults. This leaves fewer newborns appearing in each racial or ethnic group relative to the number of deliveries recorded in each group.

**Table 2:** Demographic characteristics of newborns and deliveries 2011–2019

	Deliveries				Newborns			
Group	% Total ident.	% Total pre-match	% Total matched	% Total unmatch.	% Total ident.	% Total pre-match	% Total matched	% Total unmatch.
**Region**								
South	43.5	43.7	42.1	48.4	43.1	43.1	42.1	45.1
West	23.4	23.5	20.1	33.1	23.0	23.2	20.1	29.6
Midwest	18.9	18.6	22.0	8.8	19.2	19.2	22.0	13.2
Northeast	14.2	14.2	15.8	9.8	14.7	14.6	15.8	12.1
**Managed Care Status**								
Enrolled	72.2	72.1	73.7	67.5	–	–	–	–
Not Enrolled/Other	27.8	27.9	26.3	32.5	–	–	–	–
**Age**								
20–24	30.9	31.0	31.5	29.5	–	–	–	–
25–29	29.8	29.8	30.0	29.2	–	–	–	–
30–34	19.2	19.1	19.0	19.5	–	–	–	–
35+	11.7	11.7	11.2	12.9	–	–	–	–
<20	8.3	8.4	8.2	8.8	–	–	–	–
**Sex**								
Female	99.3	99.3	99.8	97.8	48.7	48.7	48.7	48.6
Unknown	0.6	0.6	0.1	1.8	0.4	0.4	0.3	0.6
Male	0.2	0.1	0.1	0.3	50.9	50.9	51.0	50.8
**Race/Ethnicity**								
White	38.3	38.4	41.8	28.5	30.4	30.3	34.6	21.4
Hispanic	25.6	25.7	20.6	40.2	17.6	17.7	17.7	17.7
Black	21.2	21.1	22.1	18.0	17.2	17.1	19.4	12.4
Unknown/Other	6.5	6.5	6.3	7.0	28.4	28.5	21.0	44.1
Asian	3.1	3.1	3.2	2.7	2.1	2.1	2.5	1.3
Multiracial	3.0	3.0	3.3	2.0	2.6	2.6	2.9	2.2
AIAN	1.6	1.6	1.8	0.9	1.2	1.2	1.6	0.5
Hawaiian/PI	0.7	0.7	0.8	0.6	0.4	0.4	0.5	0.3

Note: Age and managed care status are not summarized for newborns. Values are percentages. Column headers are abbreviated as follows: “% Total Ident.” = Percent of total identified, “% Total Pre-match” = Percent of total before matching, “% Total Matched” = Percent of total successfully matched, “% Total Unmt.” = Percent of total unmatched

## Discussion

Our method of identifying infant–mother dyads in nationwide Medicaid data represents a substantial advancement over existing practices and provides a framework for researchers seeking to incorporate birth circumstances into studies of children’s health and the intergenerational impacts of public policy. The approach also opens new opportunities for research in maternal pharmacology. Importantly, it enables large-scale, nationwide, longitudinal analyses while safeguarding beneficiary privacy. By pooling data across time and geography, the method further facilitates the study of small and underrepresented populations that are often excluded from prior analyses because of sample size constraints.

These advances are possible because we harmonize MAX and TAF data, bridge the ICD-9 to ICD-10 transition, and address wide variation in how states enroll newborns in Medicaid and define household units. By not relying on vital records, we demonstrate that a single, flexible linking algorithm can be applied to a very large population [32]. Nevertheless, several limitations should be acknowledged. First, although we are able to identify and link newborns and deliveries across all states and years, researchers may wish to exclude specific states or state–years with particularly low linkage rates or discrepancies between Medicaid claims and NVSS counts. Second, while avoiding reliance on vital records increases generalizability and protects privacy, it also limits the number of family-level characteristics that can be observed. Third, our algorithm does not link approximately one third of Medicaid-enrolled infants to a delivery record. This reflects both data structure limitations—including incomplete or inconsistent identifiers—and coverage issues, such as instances where an infant is Medicaid-enrolled at birth but the delivery itself was not paid for by Medicaid. Improvements such as the addition of a unique family identifier, more reliable maternal identifiers, or tighter integration with birth certificate data could improve linkage success, though any such changes must be weighed carefully against privacy concerns.

Finally, while studying Medicaid beneficiaries is crucial for understanding U.S. public health, births covered by Medicaid do not perfectly mirror the demographics of all U.S. births. For example, in 2014–2016, people giving birth nationwide were approximately 53.5% non-Hispanic white, 23.1% Hispanic, and 14.8% non-Hispanic Black, compared to 38.3%, 25.6%, and 21.2%, respectively, in our Medicaid sample [33]. Moreover, Medicaid enrollment records provide only a partial measure of racial and ethnic identity [22].

In sum, nationwide longitudinal analysis of family units using Medicaid claims is both feasible and essential for advancing knowledge of maternal and child health. We hope that our algorithm will enable future work in this area and contribute meaningfully to the study of health disparities and the intergenerational effects of public policy.

## Supplementary Information

Supplementary Material 1: Appendix.

## Data Availability

Replication code is available in the repository at: doi.org/10.5281/zenodo.17088809. The Medicaid data can be obtained under a data use agreement from the CMS.

## Footnotes

1 We acknowledge that not all people delivering a child are mothers. Throughout, we analyze all Medicaid beneficiaries who receive care for delivery, including trans and non-binary parents, gestational surrogates, and other birthing people.

2 For example, we include Children’s Health Insurance Program (CHIP) and Emergency Medicaid beneficiaries.

3 For example, only beneficiaries who are enrolled for a sufficient period can be included in studies focused on healthcare utilization before and after delivery.

4 We use the term claims to refer to all claims and encounter records, specifically rows from all inpatient and other services files.

5 This restriction reflects an analytic choice intended to minimize linkage errors; researchers applying these methods may adjust the spacing interval to suit their study aims and tolerance for potential misclassification.

6 https://resdac.org/cms-data/variables/delivery-code-max-ip

7 Our algorithm is optimized to identify singleton births and deliveries, and therefore underestimates the prevalence of multiple births. Extending the algorithm to identify and link multiple births represents an area for future research.

8 Past work using diagnosis codes to identify care for newborns has identified only slightly more newborns than expected, which is also what we see when only using claims to identify newborns [16]. This arises from significant data loss in states that bill newborn care under mother’s insurance with over-coverage in other states. We prefer to identify newborns in all states even if it means that the nationwide total is higher than expected.

9 Because the number of identified newborns exceeds the number of identified deliveries, the maximum attainable match rate for newborns is 90.89%. This asymmetry in totals accounts for the higher match rate reported for deliveries compared to newborns.

10 Note that while we observe multiracial identification, we treat multiracial beneficiaries as if their race and ethnicity was unknown while linking newborns and deliveries. Table 2 reflects records observed in enrollment data rather than records used for linking.
