# Supplementary material for: Linking deliveries to newborns using nationwide Medicaid data
Source: BMC Med Res Methodol. 2025 Oct 24;25:240. doi: 10.1186/s12874-025-02688-x (PMC12553173; doi:10.1186/s12874-025-02688-x)
Supplement: Supplementary file 1 — Supplementary Material 1: Appendix. [file 12874_2025_2688_MOESM1_ESM.pdf]

Appendix

Contents

1 Data Acquisition **2**

2 Identification of Deliveries **3**

2.1 Diagnosis and Procedure Codes . . . 3

2.2 Clustering of Delivery Claims into Unique Deliveries . . . 13

2.3 Total Deliveries by State and Year . . . 15

2.4 Identifying Outliers by State/Year . . . 17

3 Identification of Newborns **19**

3.1 Diagnosis and Procedure Codes . . . 19

3.2 Total Newborns by State and Year . . . 24

3.3 Identifying Outliers by State/Year . . . 27

4 Data Cleaning **29**

4.1 Delivery Date Window . . . 29

4.2 Birth Date . . . 30

4.3 Residential Zip Code . . . 30

4.4 Facility Zip Code . . . 30

4.5 Race and Ethnicity . . . 31

4.6 Case Number . . . 31

4.7 Overall Missingness . . . 31

5 Matching **33**

5.1 Description of Matching . . . 33

5.2 Matching Rates by State . . . 33

References **37**

1 Data Acquisition

For the years 2011-2019, we acquired all of the Medicaid data available that contained information on births, deliveries, related prescriptions and services, and demographic data. CMS transitioned structures from the Medicaid Analytical Extract (MAX) data structure to the T-Msis (TAF) data structure over the course of 2014-2015, reflected in the data filed we acquired each year. In addition, the Annual Plan and Annual Provider files became available for the TAF data structure which we began acquiring in 2015. We initially acquired long-term care files for 2011, 2015, 2019 for exploratory purposes and since they were of little value to the linking, we did not acquire them for the additional years.

Table 1: Medicaid Delivery Codes Used for Identification

Year	Data Type	Data Year
2011	MAX	Personal Summary, Inpatient, Prescription Drug, Other Services, Long-term Care
2012	MAX	Personal Summary, Inpatient, Prescription Drug, Other Services
2013	MAX	Personal Summary, Inpatient, Prescription Drug, Other Services
2014	MAX	Personal Summary, Inpatient, Prescription Drug, Other Services
2014	TAF	Demographic Eligibility, Inpatient, Prescription Drug, Other Services, Annual Plan, Annual Provider
2015	MAX	Personal Summary, Inpatient, Prescription Drug, Other Services, Long-term Care
2015	TAF	Demographic Eligibility, Inpatient, Prescription Drug, Other Services, Long-term Care, Annual Plan, Annual Provider
2016	TAF	Demographic Eligibility, Inpatient, Prescription Drug, Other Services, Annual Plan, Annual Provider
2017	TAF	Demographic Eligibility, Inpatient, Prescription Drug, Other Services, Annual Plan, Annual Provider
2018	TAF	Demographic Eligibility, Inpatient, Prescription Drug, Other Services, Long-term Care, Annual Plan, Annual Provider
2019	TAF	Demographic Eligibility, Inpatient, Prescription Drug, Other Services, Annual Plan, Annual Provider

To acquire this data, we worked with the Office of Research Administration at Stanford University to submit an application to the Research Data and Assistance Center (ResDAC), the agency that assists researchers in obtaining CMS data, detailing our scope of work and rationale for acquiring each data file. We worked to revise and finalize our request through ResDAC's review processes, and with that approval, received CMS review and approval of our request. We then paid for the data and worked with the CMS approved data distributor, GDIT, to receive the files. This process took 11 months on average.

To unpack the data, the team downloaded state-year flat files and processed each file using SAS scripts provided by CMS. The team then appended each individual file into files maintained in CMS-approved cloud storage.

We accessed archived NPPES records from the National Bureau of Economics Research (National Bureau of Economic Research, n.d.). We received approval for linking NPPES records to NPIs appearing in Medicaid claims from CMS. We exported one snapshot for the NPPES registry for each year covered in our study, 2011 to 2019. When available, we used records from July. July records were unavailable for 2013 and 2019. We instead used March and April records, respectively.

In order to benchmark our identification of Medicaid deliveries and newborns from the claims data, we acquired National Vital Statistics System (NVSS) data on natality from the National Center for Health Statistics (Centers for Disease Control and Prevention, n.d.). This provides us with information on every delivery nationwide, including date of birth, birthplace, number of newborns, and payment method for delivery care. In order to use this delivery-level data, the team downloaded the yearly flat files and processed each file in R.

2 Identification of Deliveries

2.1 Diagnosis and Procedure Codes

In identifying deliveries, we first determined ICD-9, ICD-10, and CPT codes that would indicate delivery care or a delivery procedure in our claims data. Due to copyright restrictions, we do not list the specific CPT codes we used in Tables 2–4. A complete list is available from the authors upon request. We started by referencing the delivery codes used in Approach 4 in Auty et al. (2024), "Comparing approaches to identify live births using the Transformed Medicaid Statistical Information System." This approach identifies 19 ICD-10 diagnosis codes that not only identify live births in the IP and OT files but also exclude codes that are likely to indicate care for infants. This is necessary because some states allow providers to bill for infant care services using the mother's Medicaid ID for the first year of life. Using icd9data.com as a reference, we also pulled the ICD-9 code equivalents from these 19 ICD-10 codes, which resulted in a total of 28 codes. We additionally included 4 CPT codes, indicating vaginal or cesarean delivery, based on a review of the AMA's Current Procedural Terminology, Professional Edition (American Medical Association various).

We also used ICD-9 codes starting with V30 and V39 and ICD-10 codes starting with Z38 at the recommendation of colleagues at Duke University. Finally, we included an additional 9 CPT codes from the CDC's Reference Guide on Identifying Severe Maternal Morbidity. There are 17 CPT codes identified in this reference guide, 5 of which we had already included in our set of codes and 4 of which we decide to exclude. In selecting codes, we avoided codes indicating a specific condition even if that condition likely indicates that a delivery took place, and avoided codes that frequently accompany delivery not resulting in live birth. Table 2 shows our full list of 96 delivery codes.

To identify claims for delivery related care, we selected all rows from the inpatient and other services files which contained one of these 96 codes, conditional on the beneficiary being between 12 and 50 years old at the time of the claim. Age was assessed based on enrollment records for each beneficiary, identified by MSIS ID and state. We required that dates of birth be consistent across all annual enrollment records for the beneficiary, else we treated age as if it was unknown.

Table 2: Medicaid Delivery Codes Used for Identification

Code	Code Type	Description
O80	ICD-10-CM	Encounter For Full-Term Uncomplicated Delivery
O82	ICD-10-CM	Encounter For Cesarean Delivery Without Indication
Z370	ICD-10-CM	Single Live Birth
Z372	ICD-10-CM	Twins, Both Liveborn
Z373	ICD-10-CM	Twins, One Liveborn And One Stillborn
Z3750	ICD-10-CM	Multiple Births, Unspecified, All Liveborn
Z3751	ICD-10-CM	Triplets, All Liveborn
Z3752	ICD-10-CM	Quadruplets, All Liveborn
Z3753	ICD-10-CM	Quintuplets, All Liveborn
Z3754	ICD-10-CM	Sextuplets, All Liveborn
Z3759	ICD-10-CM	Other Multiple Births, All Liveborn
Z3760	ICD-10-CM	Multiple Births, Unspecified, Some Liveborn
Z3761	ICD-10-CM	Triplets, Some Liveborn
Z3762	ICD-10-CM	Quadruplets, Some Liveborn
Z3763	ICD-10-CM	Quintuplets, Some Liveborn
Z3764	ICD-10-CM	Sextuplets, Some Liveborn
Z3769	ICD-10-CM	Other Multiple Births, Some Liveborn
Z379	ICD-10-CM	Outcome Of Delivery, Unspecified
Z3800	ICD-10-CM	Single Liveborn Infant, Delivered Vaginally
Z3801	ICD-10-CM	Single Liveborn Infant, Delivered By Cesarean
Z381	ICD-10-CM	Single Liveborn Infant, Born Outside Hospital
Z382	ICD-10-CM	Single Liveborn Infant, Unspecified As To Place Of Birth
Z3830	ICD-10-CM	Twin Liveborn Infant, Delivered Vaginally
Z3831	ICD-10-CM	Twin Liveborn Infant, Delivered By Cesarean
Z384	ICD-10-CM	Twin Liveborn Infant, Born Outside Hospital
Z385	ICD-10-CM	Twin Liveborn Infant, Unspecified As To Place Of Birth
Z3861	ICD-10-CM	Triplet Liveborn Infant, Delivered Vaginally
Z3862	ICD-10-CM	Triplet Liveborn Infant, Delivered By Cesarean
Z3863	ICD-10-CM	Quadruplet Liveborn Infant, Delivered Vaginally
Z3864	ICD-10-CM	Quadruplet Liveborn Infant, Delivered By Cesarean
Z3865	ICD-10-CM	Quintuplet Liveborn Infant, Delivered Vaginally
Z3866	ICD-10-CM	Quintuplet Liveborn Infant, Delivered By Cesarean
Z3868	ICD-10-CM	Other Multiple Liveborn Infant, Delivered Vaginally
Z3869	ICD-10-CM	Other Multiple Liveborn Infant, Delivered By Cesarean
Z387	ICD-10-CM	Other Multiple Liveborn Infant, Born Outside Hospital
Z388	ICD-10-CM	Other Multiple Liveborn Infant, Unspecified As To Place Of Birth
Z390	ICD-10-CM	Encounter For Care And Examination Of Mother Immediately After Delivery
650	ICD-9-CM	Normal Delivery
66971	ICD-9-CM	Cesarean Delivery, Without Mention Of Indication, Delivered, With Or Without Mention Of Antepartum Condition
V240	ICD-9-CM	Encounter For Care And Examination Of Mother Immediately After Delivery
V270	ICD-9-CM	Outcome Of Delivery, Single Liveborn
V272	ICD-9-CM	Outcome Of Delivery, Twins, Both Liveborn
V273	ICD-9-CM	Outcome Of Delivery, Twins, One Liveborn And One Stillborn
V275	ICD-9-CM	Outcome Of Delivery, Other Multiple Birth, All Liveborn
V276	ICD-9-CM	Outcome Of Delivery, Other Multiple Birth, Some Liveborn
V279	ICD-9-CM	Outcome Of Delivery, Unspecified
V3000	ICD-9-CM	Single Liveborn, Born In Hospital, Delivered Without Mention Of Cesarean Section
V3001	ICD-9-CM	Single Liveborn, Born In Hospital, Delivered By Cesarean Section
V301	ICD-9-CM	Single Liveborn, Born Before Admission To Hospital

V302	ICD-9-CM	Single Liveborn, Born Outside Hospital And Not Hospitalized
V3100	ICD-9-CM	Twin Birth, Mate Liveborn, Born In Hospital, Delivered Without Mention Of Cesarean Section
V3101	ICD-9-CM	Twin Birth, Mate Liveborn, Born In Hospital, Delivered By Cesarean Section
V311	ICD-9-CM	Twin Birth, Mate Liveborn, Born Before Admission To Hospital
V312	ICD-9-CM	Twin Birth, Mate Liveborn, Born Outside Hospital And Not Hospitalized
V3200	ICD-9-CM	Twin Birth, Mate Stillborn, Born In Hospital, Delivered Without Mention Of Cesarean Section
V3201	ICD-9-CM	Twin Birth, Mate Stillborn, Born In Hospital, Delivered By Cesarean Section
V321	ICD-9-CM	Twin Birth, Mate Stillborn, Born Before Admission To Hospital
V322	ICD-9-CM	Twin Birth, Mate Stillborn, Born Outside Hospital And Not Hospitalized
V3300	ICD-9-CM	Twin Birth, Unspecified Whether Mate Liveborn Or Stillborn, Born In Hospital, Delivered Without Mention Of Cesarean Section
V3301	ICD-9-CM	Twin Birth, Unspecified Whether Mate Liveborn Or Stillborn, Born In Hospital, Delivered By Cesarean Section
V331	ICD-9-CM	Twin Birth, Unspecified Whether Mate Liveborn Or Stillborn, Born Before Admission To Hospital
V332	ICD-9-CM	Twin Birth, Unspecified Whether Mate Liveborn Or Stillborn, Born Outside Hospital And Not Hospitalized
V3400	ICD-9-CM	Other Multiple Birth (Three Or More), Mates All Liveborn, Born In Hospital, Delivered Without Mention Of Cesarean Section
V3401	ICD-9-CM	Other Multiple Birth (Three Or More), Mates All Liveborn, Born In Hospital, Delivered By Cesarean Section
V341	ICD-9-CM	Other Multiple Birth (Three Or More), Mates All Liveborn, Born Before Admission To Hospital
V342	ICD-9-CM	Other Multiple Birth (Three Or More), Mates All Liveborn, Born Outside Hospital And Not Hospitalized
V3500	ICD-9-CM	Other Multiple Birth (Three Or More), Mates All Still Born, Born In Hospital, Delivered Without Mention Of Cesarean Section
V3501	ICD-9-CM	Other Multiple Birth (Three Or More), Mates All Still Born, Born In Hospital, Delivered By Cesarean Section
V351	ICD-9-CM	Other Multiple Birth (Three Or More), Mates All Stillborn, Born Before Admission To Hospital
V352	ICD-9-CM	Other Multiple Birth (Three Or More), Mates All Stillborn, Born Outside Of Hospital And Not Hospitalized
V3600	ICD-9-CM	Other Multiple Birth (Three Or More), Mates Liveborn And Stillborn, Born In Hospital, Delivered Without Mention Of Cesarean Section
V3601	ICD-9-CM	Other Multiple Birth (Three Or More), Mates Liveborn And Stillborn, Born In Hospital, Delivered Without Mention Of Cesarean Section
V361	ICD-9-CM	Other Multiple Birth (Three Or More), Mates Liveborn And Stillborn, Born Before Admission To Hospital
V362	ICD-9-CM	Other Multiple Birth (Three Or More), Mates Liveborn And Stillborn, Born Outside Hospital And Not Hospitalized
V3700	ICD-9-CM	Other Multiple Birth (Three Or More), Unspecified Whether Mates Liveborn Or Stillborn, Born In Hospital, Delivered Without Mention Of Cesarean Section
V3701	ICD-9-CM	Other Multiple Birth (Three Or More), Unspecified Whether Mates Liveborn Or Stillborn, Born In Hospital, Delivered By Cesarean Section
V371	ICD-9-CM	Other Multiple Birth (Three Or More), Unspecified Whether Mates Liveborn Or Stillborn, Born Before Admission To Hospital
V372	ICD-9-CM	Other Multiple Birth (Three Or More), Unspecified Whether Mates Liveborn Or Stillborn, Born Outside Of Hospital
V3900	ICD-9-CM	Liveborn, Unspecified Whether Single, Twin Or Multiple, Born In Hospital, Delivered Without Mention Of Cesarean Section

V3901	ICD-9-CM	Liveborn, Unspecified Whether Single, Twin Or Multiple, Born In Hospital, Delivered By Cesarean Section
V391	ICD-9-CM	Liveborn, Unspecified Whether Single, Twin Or Multiple, Born Before Admission To Hospital
V392	ICD-9-CM	Liveborn, Unspecified Whether Single, Twin Or Multiple, Born Outside Hospital And Not Hospitalized

Table 3 is our list of twins or multiple birth codes that we use to exclude certain delivery claims from our analysis. We first identify all deliveries by clustering claims for delivery related care, then exclude any clusters containing claims with the following codes. While this linking algorithm could be extended to multiple births, our focus in building this algorithm is linking single births.

Table 3: Medicaid Delivery Codes for Twins

Code	Code Type	Description
O30	ICD-10-CM	Multiple Gestation
O300	ICD-10-CM	Twin Pregnancy
O3000	ICD-10-CM	Twin Pregnancy, Unspecified Number Of Placenta And Unspecified Number Of Amniotic Sacs
O30001	ICD-10-CM	Twin Pregnancy, Unspecified Number Of Placenta And Unspecified Number Of Amniotic Sacs, First Trimester
O30002	ICD-10-CM	Twin Pregnancy, Unspecified Number Of Placenta And Unspecified Number Of Amniotic Sacs, Second Trimester
O30003	ICD-10-CM	Twin Pregnancy, Unspecified Number Of Placenta And Unspecified Number Of Amniotic Sacs, Third Trimester
O30009	ICD-10-CM	Twin Pregnancy, Unspecified Number Of Placenta And Unspecified Number Of Amniotic Sacs, Unspecified Trimester
O3001	ICD-10-CM	Twin Pregnancy, Monochorionic/Monoamniotic
O30011	ICD-10-CM	Twin Pregnancy, Monochorionic/Monoamniotic, First Trimester
O30012	ICD-10-CM	Twin Pregnancy, Monochorionic/Monoamniotic, Second Trimester
O30013	ICD-10-CM	Twin Pregnancy, Monochorionic/Monoamniotic, Third Trimester
O30019	ICD-10-CM	Twin Pregnancy, Monochorionic/Monoamniotic, Unspecified Trimester
O3002	ICD-10-CM	Conjoined Twin Pregnancy
O30021	ICD-10-CM	Conjoined Twin Pregnancy, First Trimester
O30022	ICD-10-CM	Conjoined Twin Pregnancy, Second Trimester
O30023	ICD-10-CM	Conjoined Twin Pregnancy, Third Trimester
O30029	ICD-10-CM	Conjoined Twin Pregnancy, Unspecified Trimester
O3003	ICD-10-CM	Twin Pregnancy, Monochorionic/Diamniotic
O30031	ICD-10-CM	Twin Pregnancy, Monochorionic/Diamniotic, First Trimester
O30032	ICD-10-CM	Twin Pregnancy, Monochorionic/Diamniotic, Second Trimester
O30033	ICD-10-CM	Twin Pregnancy, Monochorionic/Diamniotic, Third Trimester
O30039	ICD-10-CM	Twin Pregnancy, Monochorionic/Diamniotic, Unspecified Trimester
O3004	ICD-10-CM	Twin Pregnancy, Dichorionic/Diamniotic
O30041	ICD-10-CM	Twin Pregnancy, Dichorionic/Diamniotic, First Trimester
O30042	ICD-10-CM	Twin Pregnancy, Dichorionic/Diamniotic, Second Trimester
O30043	ICD-10-CM	Twin Pregnancy, Dichorionic/Diamniotic, Third Trimester
O30049	ICD-10-CM	Twin Pregnancy, Dichorionic/Diamniotic, Unspecified Trimester
O3009	ICD-10-CM	Twin Pregnancy, Unable To Determine Number Of Placenta And Number Of Amniotic Sacs
O30091	ICD-10-CM	Twin Pregnancy, Unable To Determine Number Of Placenta And Number Of Amniotic Sacs, First Trimester
O30092	ICD-10-CM	Twin Pregnancy, Unable To Determine Number Of Placenta And Number Of Amniotic Sacs, Second Trimester
O30093	ICD-10-CM	Twin Pregnancy, Unable To Determine Number Of Placenta And Number Of Amniotic Sacs, Third Trimester
O30099	ICD-10-CM	Twin Pregnancy, Unable To Determine Number Of Placenta And Number Of Amniotic Sacs, Unspecified Trimester
O301	ICD-10-CM	Triplet Pregnancy
O3010	ICD-10-CM	Triplet Pregnancy, Unspecified Number Of Placenta And Unspecified Number Of Amniotic Sacs

O30101	ICD-10-CM	Triplet Pregnancy, Unspecified Number Of Placenta And Unspecified Number Of Amniotic Sacs, First Semester
O30102	ICD-10-CM	Triplet Pregnancy, Unspecified Number Of Placenta And Unspecified Number Of Amniotic Sacs, Second Semester
O30103	ICD-10-CM	Triplet Pregnancy, Unspecified Number Of Placenta And Unspecified Number Of Amniotic Sacs, Third Trimester
O30109	ICD-10-CM	Triplet Pregnancy, Unspecified Number Of Placenta And Unspecified Number Of Amniotic Sacs, Unspecified Trimester
O3011	ICD-10-CM	Triplet Pregnancy With Two Or More Monochorionic Fetuses
O30111	ICD-10-CM	Triplet Pregnancy With Two Or More Monochorionic Fetuses, First Trimester
O30112	ICD-10-CM	Triplet Pregnancy With Two Or More Monochorionic Fetuses, Second Trimester
O30113	ICD-10-CM	Triplet Pregnancy With Two Or More Monochorionic Fetuses, Third Trimester
O30119	ICD-10-CM	Triplet Pregnancy With Two Or More Monochorionic Fetuses, Unspecified Trimester
O3012	ICD-10-CM	Triplet Pregnancy With Two Or More Monoamniotic Fetuses
O30121	ICD-10-CM	Triplet Pregnancy With Two Or More Monoamniotic Fetuses, First Trimester
O30122	ICD-10-CM	Triplet Pregnancy With Two Or More Monoamniotic Fetuses, Second Trimester
O30123	ICD-10-CM	Triplet Pregnancy With Two Or More Monoamniotic Fetuses, Third Trimester
O30129	ICD-10-CM	Triplet Pregnancy With Two Or More Monoamniotic Fetuses, Unspecified Trimester
O3013	ICD-10-CM	Triplet Pregnancy, Trichorionic/Triamniotic
O30131	ICD-10-CM	Triplet Pregnancy, Trichorionic/Triamniotic, First Trimester
O30132	ICD-10-CM	Triplet Pregnancy, Trichorionic/Triamniotic, Second Trimester
O30133	ICD-10-CM	Triplet Pregnancy, Trichorionic/Triamniotic, Third Trimester
O30139	ICD-10-CM	Triplet Pregnancy, Trichorionic/Triamniotic, Unspecified Trimester
O3019	ICD-10-CM	Triplet Pregnancy, Unable To Determine Number Of Placenta And Number Of Amniotic Sacs
O30191	ICD-10-CM	Triplet Pregnancy, Unable To Determine Number Of Placenta And Number Of Amniotic Sacs, First Trimester
O30192	ICD-10-CM	Triplet Pregnancy, Unable To Determine Number Of Placenta And Number Of Amniotic Sacs, Second Trimester
O30193	ICD-10-CM	Triplet Pregnancy, Unable To Determine Number Of Placenta And Number Of Amniotic Sacs, Third Trimester
O30199	ICD-10-CM	Triplet Pregnancy, Unable To Determine Number Of Placenta And Number Of Amniotic Sacs, Unspecified Trimester
O302	ICD-10-CM	Quadruplet Pregnancy
O3020	ICD-10-CM	Quadruplet Pregnancy, Unspecified Number Of Placenta And Unspecified Number Of Amniotic Sacs
O30201	ICD-10-CM	Quadruplet Pregnancy, Unspecified Number Of Placenta And Unspecified Number Of Amniotic Sacs, First Trimester
O30202	ICD-10-CM	Quadruplet Pregnancy, Unspecified Number Of Placenta And Unspecified Number Of Amniotic Sacs, Second Trimester
O30203	ICD-10-CM	Quadruplet Pregnancy, Unspecified Number Of Placenta And Unspecified Number Of Amniotic Sacs, Third Trimester
O30209	ICD-10-CM	Quadruplet Pregnancy, Unspecified Number Of Placenta And Unspecified Number Of Amniotic Sacs, Unspecified Trimester
O3021	ICD-10-CM	Quadruplet Pregnancy With Two Or More Monochorionic Fetuses
O30211	ICD-10-CM	Quadruplet Pregnancy With Two Or More Monochorionic Fetuses, First Trimester
O30212	ICD-10-CM	Quadruplet Pregnancy With Two Or More Monochorionic Fetuses, Second Trimester
O30213	ICD-10-CM	Quadruplet Pregnancy With Two Or More Monochorionic Fetuses, Third Trimester

O30219	ICD-10-CM	Quadruplet Pregnancy With Two Or More Monochorionic Fetuses, Unspecified Trimester
O3022	ICD-10-CM	Quadruplet Pregnancy With Two Or More Monoamniotic Fetuses
O30221	ICD-10-CM	Quadruplet Pregnancy With Two Or More Monoamniotic Fetuses, First Trimester
O30222	ICD-10-CM	Quadruplet Pregnancy With Two Or More Monoamniotic Fetuses, Second Trimester
O30223	ICD-10-CM	Quadruplet Pregnancy With Two Or More Monoamniotic Fetuses, Third Trimester
O30229	ICD-10-CM	Quadruplet Pregnancy With Two Or More Monoamniotic Fetuses, Unspecified Trimester
O3023	ICD-10-CM	Quadruplet Pregnancy, Quadrachorionic/Quadra-Amniotic
O30231	ICD-10-CM	Quadruplet Pregnancy, Quadrachorionic/Quadra-Amniotic, First Trimester
O30232	ICD-10-CM	Quadruplet Pregnancy, Quadrachorionic/Quadra-Amniotic, Second Trimester
O30233	ICD-10-CM	Quadruplet Pregnancy, Quadrachorionic/Quadra-Amniotic, Third Trimester
O30239	ICD-10-CM	Quadruplet Pregnancy, Quadrachorionic/Quadra-Amniotic, Unspecified Trimester
O3029	ICD-10-CM	Quadruplet Pregnancy, Unable To Determine Number Of Placenta And Number Of Amniotic Sacs
O30291	ICD-10-CM	Quadruplet Pregnancy, Unable To Determine Number Of Placenta And Number Of Amniotic Sacs, First Trimester
O30292	ICD-10-CM	Quadruplet Pregnancy, Unable To Determine Number Of Placenta And Number Of Amniotic Sacs, Second Trimester
O30293	ICD-10-CM	Quadruplet Pregnancy, Unable To Determine Number Of Placenta And Number Of Amniotic Sacs, Third Trimester
O30299	ICD-10-CM	Quadruplet Pregnancy, Unable To Determine Number Of Placenta And Number Of Amniotic Sacs, Unspecified Trimester
O308	ICD-10-CM	Other Specified Multiple Gestation
O3080	ICD-10-CM	Other Specified Multiple Gestation, Unspecified Number Of Placenta And Unspecified Number Of Amniotic Sacs
O30801	ICD-10-CM	Other Specified Multiple Gestation, Unspecified Number Of Placenta And Unspecified Number Of Amniotic Sacs, First Trimester
O30802	ICD-10-CM	Other Specified Multiple Gestation, Unspecified Number Of Placenta And Unspecified Number Of Amniotic Sacs, Second Trimester
O30803	ICD-10-CM	Other Specified Multiple Gestation, Unspecified Number Of Placenta And Unspecified Number Of Amniotic Sacs, Third Trimester
O30809	ICD-10-CM	Other Specified Multiple Gestation, Unspecified Number Of Placenta And Unspecified Number Of Amniotic Sacs, Unspecified Trimester
O3081	ICD-10-CM	Other Specified Multiple Gestation With Two Or More Monochorionic Fetuses
O30811	ICD-10-CM	Other Specified Multiple Gestation With Two Or More Monochorionic Fetuses, First Trimester
O30812	ICD-10-CM	Other Specified Multiple Gestation With Two Or More Monochorionic Fetuses, Second Trimester
O30813	ICD-10-CM	Other Specified Multiple Gestation With Two Or More Monochorionic Fetuses, Third Trimester
O30819	ICD-10-CM	Other Specified Multiple Gestation With Two Or More Monochorionic Fetuses, Unspecified Trimester
O3082	ICD-10-CM	Other Specified Multiple Gestation With Two Or More Monoamniotic Fetuses
O30821	ICD-10-CM	Other Specified Multiple Gestation With Two Or More Monoamniotic Fetuses, First Trimester
O30822	ICD-10-CM	Other Specified Multiple Gestation With Two Or More Monoamniotic Fetuses, Second Trimester
O30823	ICD-10-CM	Other Specified Multiple Gestation With Two Or More Monoamniotic Fetuses, Third Trimester

O30829	ICD-10-CM	Other Specified Multiple Gestation With Two Or More Monoamniotic Fetuses, Unspecified Trimester
O3083	ICD-10-CM	Other Specified Multiple Gestation, Number Of Chorions And Amnions Are Both Equal To The Number Of Fetuses
O30831	ICD-10-CM	Other Specified Multiple Gestation, Number Of Chorions And Amnions Are Both Equal To The Number Of Fetuses, First Trimester
O30832	ICD-10-CM	Other Specified Multiple Gestation, Number Of Chorions And Amnions Are Both Equal To The Number Of Fetuses, Second Trimester
O30833	ICD-10-CM	Other Specified Multiple Gestation, Number Of Chorions And Amnions Are Both Equal To The Number Of Fetuses, Third Trimester
O30839	ICD-10-CM	Other Specified Multiple Gestation, Number Of Chorions And Amnions Are Both Equal To The Number Of Fetuses, Unspecified Trimester
O3089	ICD-10-CM	Other Specified Multiple Gestation, Unable To Determine Number Of Placenta And Number Of Amniotic Sacs
O30891	ICD-10-CM	Other Specified Multiple Gestation, Unable To Determine Number Of Placenta And Number Of Amniotic Sacs, First Trimester
O30892	ICD-10-CM	Other Specified Multiple Gestation, Unable To Determine Number Of Placenta And Number Of Amniotic Sacs, Second Trimester
O30893	ICD-10-CM	Other Specified Multiple Gestation, Unable To Determine Number Of Placenta And Number Of Amniotic Sacs, Third Trimester
O30899	ICD-10-CM	Other Specified Multiple Gestation, Unable To Determine Number Of Placenta And Number Of Amniotic Sacs, Unspecified Trimester
O309	ICD-10-CM	Multiple Gestation, Unspecified
O3090	ICD-10-CM	Multiple Gestation, Unspecified, Unspecified Trimester
O3091	ICD-10-CM	Multiple Gestation, Unspecified, First Trimester
O3092	ICD-10-CM	Multiple Gestation, Unspecified, Second Trimester
O3093	ICD-10-CM	Multiple Gestation, Unspecified, Third Trimester
Z372	ICD-10-CM	Twins, Both Liveborn
Z3750	ICD-10-CM	Multiple Births, Unspecified, All Liveborn
Z3751	ICD-10-CM	Triplets, All Liveborn
Z3752	ICD-10-CM	Quadruplets, All Liveborn
Z3753	ICD-10-CM	Quintuplets, All Liveborn
Z3754	ICD-10-CM	Sextuplets, All Liveborn
Z3759	ICD-10-CM	Other Multiple Births, All Liveborn
Z3760	ICD-10-CM	Multiple Births, Unspecified, Some Liveborn
Z3761	ICD-10-CM	Triplets, Some Liveborn
Z3762	ICD-10-CM	Quadruplets, Some Liveborn
Z3763	ICD-10-CM	Quintuplets, Some Liveborn
Z3764	ICD-10-CM	Sextuplets, Some Liveborn
Z3769	ICD-10-CM	Other Multiple Births, Some Liveborn
Z3830	ICD-10-CM	Twin Liveborn Infant, Delivered Vaginally
Z3831	ICD-10-CM	Twin Liveborn Infant, Delivered By Cesarean
Z384	ICD-10-CM	Twin Liveborn Infant, Born Outside Hospital
Z3861	ICD-10-CM	Triplet Liveborn Infant, Delivered Vaginally
Z3862	ICD-10-CM	Triplet Liveborn Infant, Delivered By Cesarean
Z3863	ICD-10-CM	Quadruplet Liveborn Infant, Delivered Vaginally
Z3864	ICD-10-CM	Quadruplet Liveborn Infant, Delivered By Cesarean
Z3865	ICD-10-CM	Quintuplet Liveborn Infant, Delivered Vaginally
Z3866	ICD-10-CM	Quintuplet Liveborn Infant, Delivered By Cesarean
Z3868	ICD-10-CM	Other Multiple Liveborn Infant, Delivered Vaginally
Z3869	ICD-10-CM	Other Multiple Liveborn Infant, Delivered By Cesarean
Z387	ICD-10-CM	Other Multiple Liveborn Infant, Born Outside Hospital
Z388	ICD-10-CM	Other Multiple Liveborn Infant, Unspecified As To Place Of Birth
BY4GZZZ	ICD-10-PCS	Ultrasonography Of Third Trimester, Multiple Gestation

651	ICD-9-CM	Multiple Gestation
6510	ICD-9-CM	Twin Pregnancy
65100	ICD-9-CM	Twin Pregnancy, Unspecified As To Episode Of Care Or Not Applicable
65101	ICD-9-CM	Twin Pregnancy, Delivered, With Or Without Mention Of Antepartum Condition
65103	ICD-9-CM	Twin Pregnancy, Antepartum Condition Or Complication
6511	ICD-9-CM	Triplet Pregnancy
65110	ICD-9-CM	Triplet Pregnancy, Unspecified As To Episode Of Care Or Not Applicable
65111	ICD-9-CM	Triplet Pregnancy, Delivered, With Or Without Mention Of Antepartum Condition
65113	ICD-9-CM	Triplet Pregnancy, Antepartum Condition Or Complication
6512	ICD-9-CM	Quadruplet Pregnancy
65120	ICD-9-CM	Quadruplet Pregnancy, Unspecified As To Episode Of Care Or Not Applicable
65121	ICD-9-CM	Quadruplet Pregnancy, Delivered, With Or Without Mention Of Antepartum Condition
65123	ICD-9-CM	Quadruplet Pregnancy, Antepartum Condition Or Complication
6513	ICD-9-CM	Twin Pregnancy With Fetal Loss And Retention Of One Fetus
65130	ICD-9-CM	Twin Pregnancy With Fetal Loss And Retention Of One Fetus, Unspecified As To Episode Of Care Or Not Applicable
6514	ICD-9-CM	Triplet Pregnancy With Fetal Loss And Retention Of One Or More Fetus(es)
65140	ICD-9-CM	Triplet Pregnancy With Fetal Loss And Retention Of One Or More Fetus(es), Unspecified As To Episode Of Care Or Not Applicable
65141	ICD-9-CM	Triplet Pregnancy With Fetal Loss And Retention Of One Or More Fetus(es), Delivered, With Or Without Mention Of Antepartum Condition
65143	ICD-9-CM	Triplet Pregnancy With Fetal Loss And Retention Of One Or More Fetus(es), Antepartum Condition Or Complication
6515	ICD-9-CM	Quadruplet Pregnancy With Fetal Loss And Retention Of One Or More Fetus(es)
65150	ICD-9-CM	Quadruplet Pregnancy With Fetal Loss And Retention Of One Or More Fetus(es), Unspecified As To Episode Of Care Or Not Applicable
65151	ICD-9-CM	Quadruplet Pregnancy With Fetal Loss And Retention Of One Or More Fetus(es), Delivered, With Or Without Mention Of Antepartum Condition
65153	ICD-9-CM	Quadruplet Pregnancy With Fetal Loss And Retention Of One Or More Fetus(es), Antepartum Condition Or Complication
6516	ICD-9-CM	Other Multiple Pregnancy With Fetal Loss And Retention Of One Or More Fetus(es)
65160	ICD-9-CM	Other Multiple Pregnancy With Fetal Loss And Retention Of One Or More Fetus(es), Unspecified As To Episode Of Care Or Not Applicable
65161	ICD-9-CM	Other Multiple Pregnancy With Fetal Loss And Retention Of One Or More Fetus(es), Delivered, With Or Without Mention Of Antepartum Condition
65163	ICD-9-CM	Other Multiple Pregnancy With Fetal Loss And Retention Of One Or More Fetus(es), Antepartum Condition Or Complication
6517	ICD-9-CM	Multiple Gestation Following (Elective) Fetal Reduction
65170	ICD-9-CM	Multiple Gestation Following (Elective) Fetal Reduction, Unspecified As To Episode Of Care Or Not Applicable
65171	ICD-9-CM	Multiple Gestation Following (Elective) Fetal Reduction,Delivered, With Or Without Mention Of Antepartum Condition
65173	ICD-9-CM	Multiple Gestation Following (Elective) Fetal Reduction, Antepartum Condition Or Complication
6518	ICD-9-CM	Other Specified Multiple Gestation
65180	ICD-9-CM	Other Specified Multiple Gestation, Unspecified As To Episode Of Care Or Not Applicable
65181	ICD-9-CM	Other Specified Multiple Gestation, Delivered, With Or Without Mention Of Antepartum Condition

65183	ICD-9-CM	Other Specified Multiple Gestation, Antepartum Condition Or Complication
6519	ICD-9-CM	Unspecified Multiple Gestation
65190	ICD-9-CM	Unspecified Multiple Gestation, Unspecified As To Episode Of Care Or Not Applicable
65191	ICD-9-CM	Unspecified Multiple Gestation, Delivered, With Or Without Mention Of Antepartum Condition
65193	ICD-9-CM	Unspecified Multiple Gestation, Antepartum Condition Or Complication
6781	ICD-9-CM	Fetal Conjoined Twins
7594	ICD-9-CM	Conjoined Twins
V272	ICD-9-CM	Outcome Of Delivery, Twins, Both Liveborn
V275	ICD-9-CM	Outcome Of Delivery, Other Multiple Birth, All Liveborn
V276	ICD-9-CM	Outcome Of Delivery, Other Multiple Birth, Some Liveborn
V3101	ICD-9-CM	Twin Birth, Mate Liveborn, Born In Hospital, Delivered By Cesarean Section
V311	ICD-9-CM	Twin Birth, Mate Liveborn, Born Before Admission To Hospital
V312	ICD-9-CM	Twin Birth, Mate Liveborn, Born Outside Hospital And Not Hospitalized
V3300	ICD-9-CM	Twin Birth, Unspecified Whether Mate Liveborn Or Stillborn, Born In Hospital, Delivered Without Mention Of Cesarean Section
V3301	ICD-9-CM	Twin Birth, Unspecified Whether Mate Liveborn Or Stillborn, Born In Hospital, Delivered By Cesarean Section
V331	ICD-9-CM	Twin Birth, Unspecified Whether Mate Liveborn Or Stillborn, Born Before Admission To Hospital
V332	ICD-9-CM	Twin Birth, Unspecified Whether Mate Liveborn Or Stillborn, Born Outside Hospital And Not Hospitalized
V3400	ICD-9-CM	Other Multiple Birth (Three Or More), Mates All Liveborn, Born In Hospital, Delivered Without Mention Of Cesarean Section
V3401	ICD-9-CM	Other Multiple Birth (Three Or More), Mates All Liveborn, Born In Hospital, Delivered By Cesarean Section
V341	ICD-9-CM	Other Multiple Birth (Three Or More), Mates All Liveborn, Born Before Admission To Hospital
V342	ICD-9-CM	Other Multiple Birth (Three Or More), Mates All Liveborn, Born Outside Hospital And Not Hospitalized
V3600	ICD-9-CM	Other Multiple Birth (Three Or More), Mates Liveborn And Stillborn, Born In Hospital, Delivered Without Mention Of Cesarean Section
V3601	ICD-9-CM	Other Multiple Birth (Three Or More), Mates Liveborn And Stillborn, Born In Hospital, Delivered Without Mention Of Cesarean Section
V361	ICD-9-CM	Other Multiple Birth (Three Or More), Mates Liveborn And Stillborn, Born Before Admission To Hospital
V362	ICD-9-CM	Other Multiple Birth (Three Or More), Mates Liveborn And Stillborn, Born Outside Hospital And Not Hospitalized
V3700	ICD-9-CM	Other Multiple Birth (Three Or More), Unspecified Whether Mates Liveborn Or Stillborn, Born In Hospital, Delivered Without Mention Of Cesarean Section
V3701	ICD-9-CM	Other Multiple Birth (Three Or More), Unspecified Whether Mates Liveborn Or Stillborn, Born In Hospital, Delivered By Cesarean Section
V371	ICD-9-CM	Other Multiple Birth (Three Or More), Unspecified Whether Mates Liveborn Or Stillborn, Born Before Admission To Hospital
V372	ICD-9-CM	Other Multiple Birth (Three Or More), Unspecified Whether Mates Liveborn Or Stillborn, Born Outside Of Hospital

2.2 Clustering of Delivery Claims into Unique Deliveries

After identifying all of our delivery-related claims using our 96 delivery codes, we have a dataset of 42,364,520 claims associated with 12,145,622 unique beneficiaries across all states and years. Our goal in using a clustering algorithm is to determine which of these claims cover care for which deliveries, given that many beneficiaries had multiple children during this nine year period.

In order to do this, we implement a hierarchical clustering algorithm with a centroid linkage method. Centroid linkage in hierarchical clustering assures a set minimum distance between the centroids of two distinct clusters. We use service begin dates from all of our claims and a minimum centroid distance of 270 days (approximately 9 months) to determine our clusters.

Before we conduct this clustering algorithm, we filter out any claims that may have codes relating to still births (65641, V271, Z371, V274, Z374, V277, Z377). We also filter out any claims that have service end dates greater than 30 days after their respective service begin dates or whose service end dates are before their respective service begin dates.

Figure 2.1: Distribution of Mean Centroid Distance between Clusters

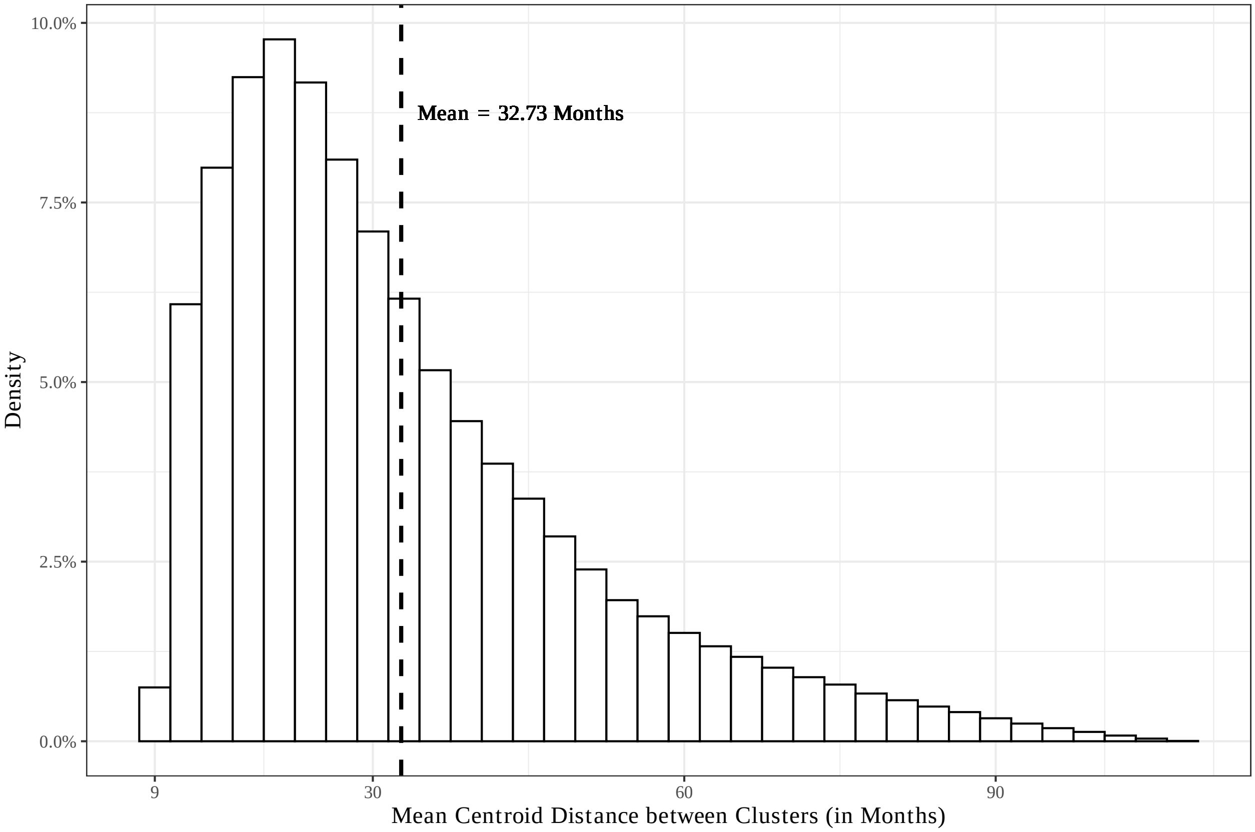

As we can see from the Figure 2.1 above, by definition, the distribution of mean centroid distance between clusters for a given beneficiary cannot be smaller than 270 days or approximately 9 months. The mean of this distribution is 997.13 days. In other words, for any beneficiary with more than one delivery in our data, we estimate that deliveries are approximately 2.73 years apart on average.

The distribution of cluster sizes per delivery can be seen in Figure 2.2. For 21.1% of deliveries we observe exactly one claim. However, the median number of claims per delivery is 2.

After applying this clustering method, we identify a total of 16,146,191 deliveries among 12,145,622 unique beneficiaries. This means that individuals in our universe of identified deliveries have, on average, 1.33 deliveries covered under Medicaid from 2011-2019.

Figure 2.2: Distribution of Delivery Cluster Sizes

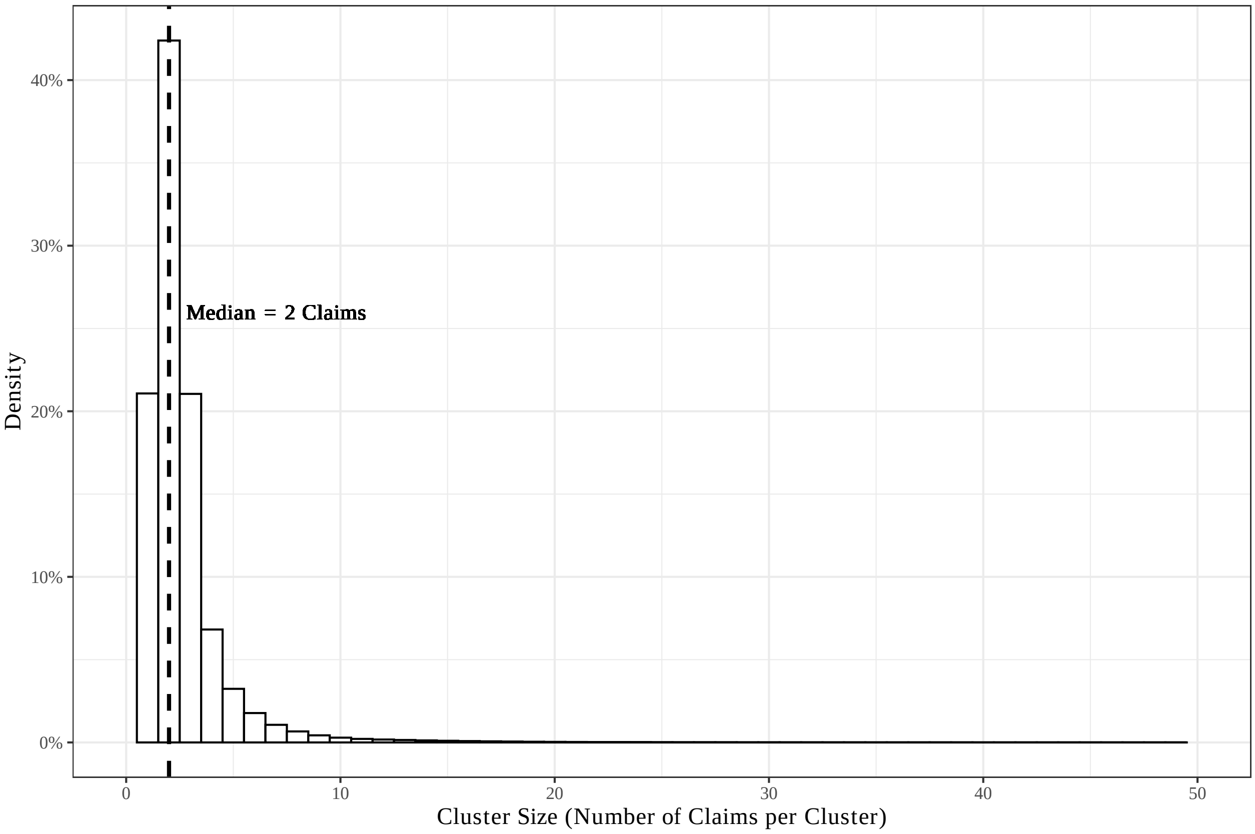

2.3 Total Deliveries by State and Year

After identifying claims using our 96 delivery codes and implementing our clustering algorithm, we identify 9.28% more deliveries compared to our NVSS benchmark.

Our NVSS benchmark was generated using data we received from the CDC's National Vital Statistics System on deliveries covered by Medicaid in each state, year, and month, as discussed in Appendix 1. Each year's worth of data includes information on each delivery, including state, delivery date, and payment method for the delivery procedure and care. The possible values for this payment method variable are Medicaid, private insurance, self pay, or other/unknown. Before we were able to calculate the number of deliveries financed by Medicaid in each state year, significant data cleaning and imputation was required. The payer variable that indicates Medicaid coverage for deliveries was missing, either partially or entirely, for some states from 2011-2015. For state/years where this variable was missing for up to 25% of the data, we employed a random proportional imputation method that uses the proportion of Medicaid coverage for deliveries without missingness for each respective state to be applied to the missing data. For state/years where this payer variable is missing at a rate higher than 25%, we applied a linear regression imputation model at the state/year level to predict the proportion of Medicaid coverage: $Medicaid\,Coverage\,Frequency_i \sim Year + State_i$. Payer records were complete for NVSS data from 2016-2019.

Figure 2.3 shows how our identified CMS deliveries compare to our number of NVSS deliveries by state and year. Each boxplot, shown at the state/year level, explains the spread of our percent differences at the state/year/month level. Our process for identifying deliveries yields a total number of deliveries that is close to what we observe in NVSS records across all years for most states. However, in Massachusetts, Maryland, Hawaii, Pennsylvania, New Mexico, Texas, Virginia, and Wyoming, we are identifying more deliveries than expected across all or nearly all years. In Nevada and Alabama, we identify fewer deliveries than expected in some years, and more in others. These differences may be a reflection of deficiencies in our identification process. There may also be errors in our NVSS benchmark, particularly for 2011-2015, given that we are partially or entirely missing the payer variable for certain states.

Figure 2.3: Deliveries by State and Year

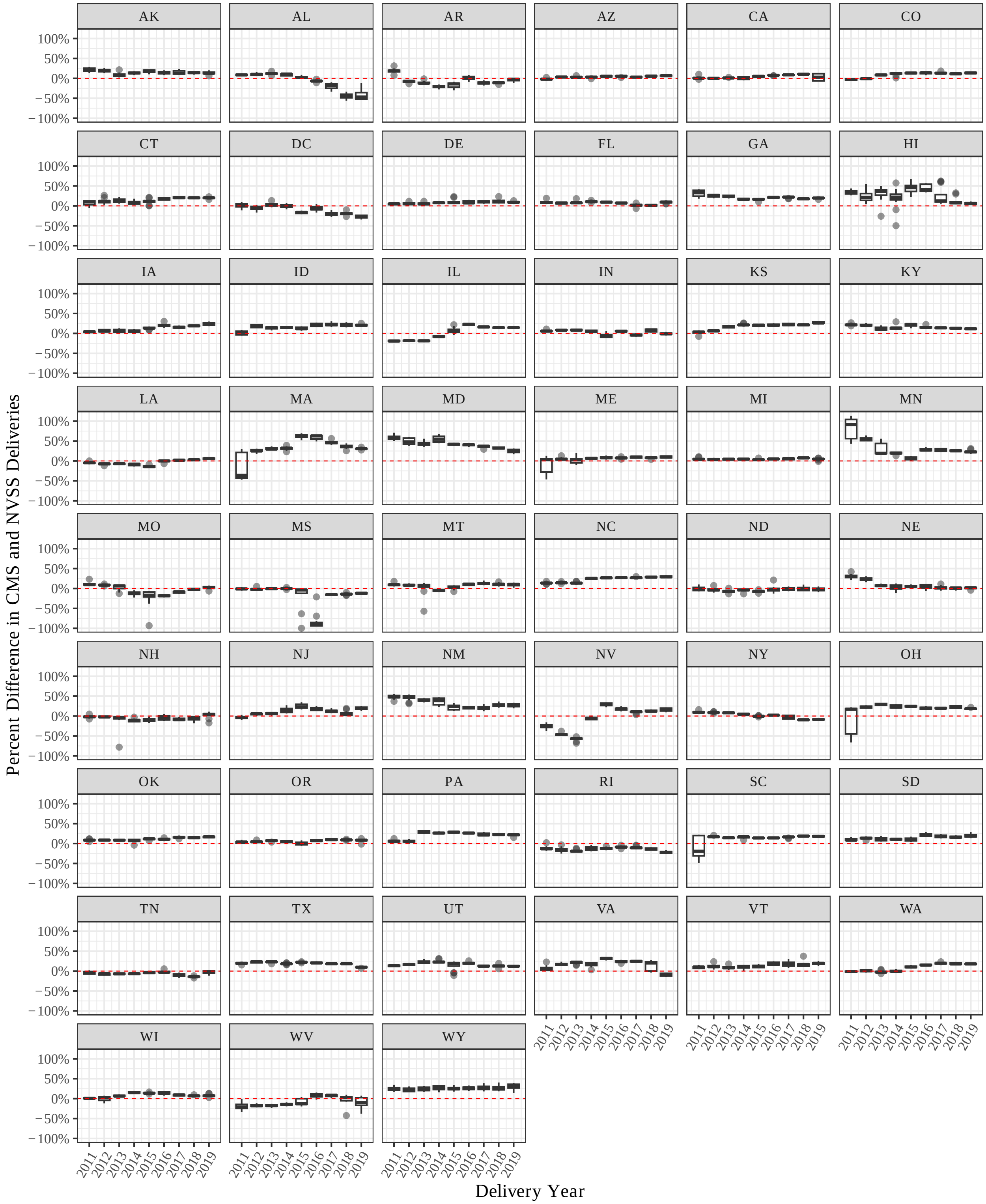

2.4 Identifying Outliers by State/Year

Figure 2.4 shows the distribution of the percent difference between he number of our identified deliveries and the number of NVSS deliveries by state and year. The dotted lines indicate the 5th percentile and 95th percentile. Our 5th percentile is a -16.78% difference and our 95th percentile is a 31.20% difference.

Figure 2.4: Percent Difference of CMS and NVSS Deliveries by State/Year

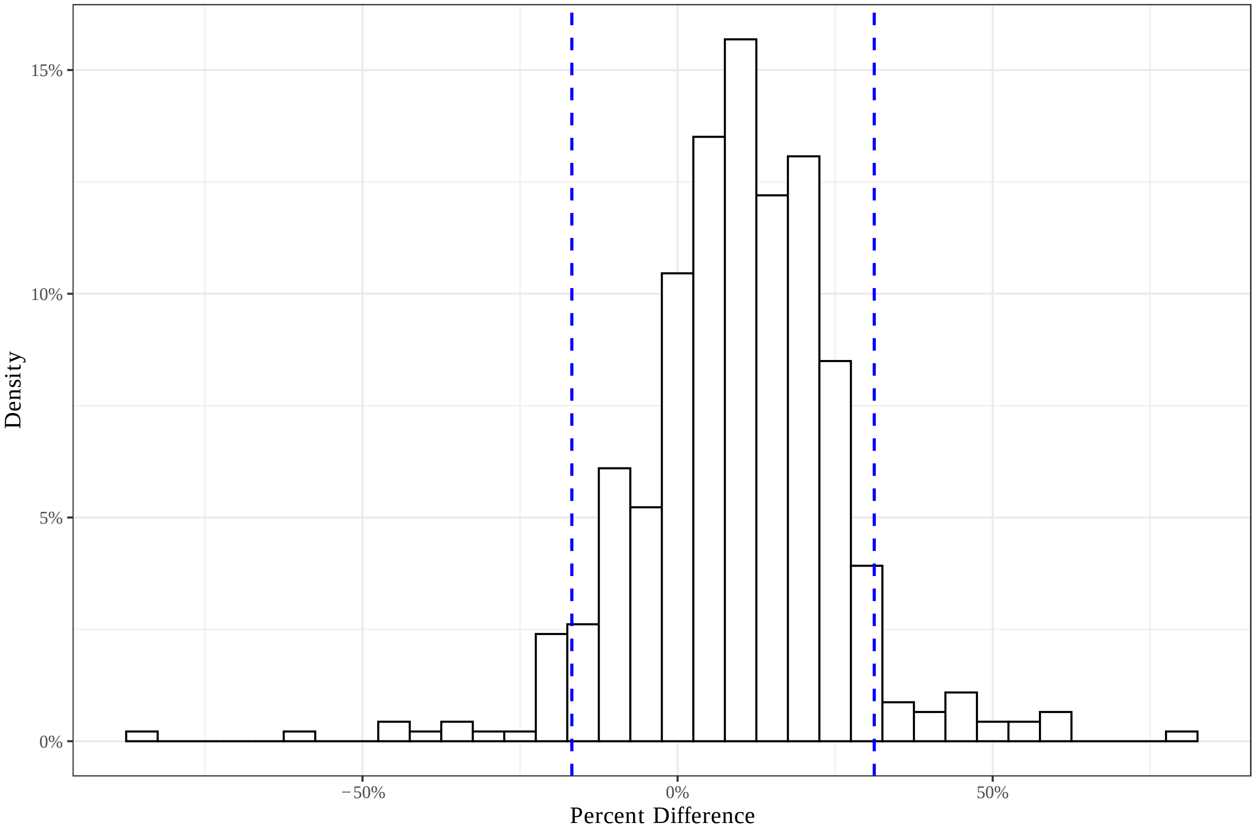

Figure 2.5 presents percent differences by state and year, indicating state/year combinations that do not fall in the middle 90% of our distribution of percent differences. We often observe these outliers in the same state across successive years. In a given state, the red points indicate years for which our percent difference of CMS deliveries versus NVSS deliveries falls above or below the middle 90% of our distribution of percent differences. Green points indicate years for which our percent differences falls within the middle 90% of our distribution.

Figure 2.5: Percent Difference of CMS and NVSS Deliveries by State/Year - Outliers

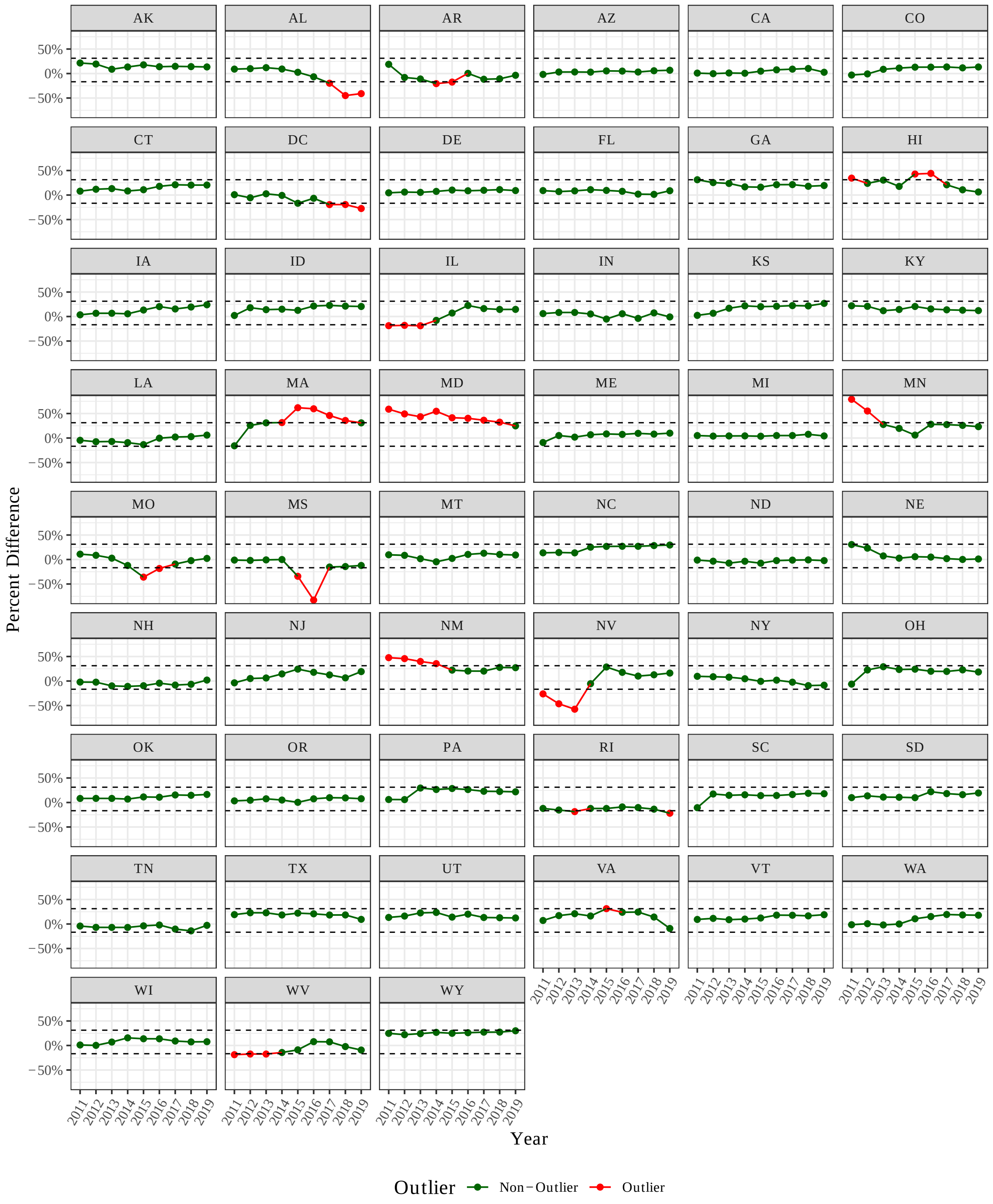

3 Identification of Newborns

3.1 Diagnosis and Procedure Codes

Table 4 describes the ICD-9, ICD-10, and CPT codes we use to initially identify newborns in our claims data. ICD-9 codes were drawn from the set of CMS codes used to identify newborns in MAX data. TAF data (which largely includes ICD-10 diagnoses) does not include a variable indicating newborn status. Instead, we identified equivalent ICD-10 codes using icd10data.com. We additionally drew on codes identified by an original review of the AMA's Current Procedural Terminology, Professional Edition (American Medical Association various). We avoided codes that indicate a specific condition, even if those conditions are specific to newborns, to avoid generating a cohort that is marginally more or less likely to include individuals on the basis of their health status.

To identify claims for care for newborns, we selected all rows from the inpatient and other services files which contained one of these 74 codes, conditional on service begin date of the claim being up to 7 days after a newborn's date of birth. In other words, we exclude any claims more than 7 days after a newborn's date of birth. We required that dates of birth be consistent across all annual enrollment records for the beneficiary, else we treated age as if it was unknown.

Table 4: Medicaid Newborn Codes Used for Identification

Code Type	Code	Description
ICD-10-CM	Z38	Liveborn Infants According To Place Of Birth And Type Of Delivery
ICD-10-CM	Z380	Single Liveborn Infant, Born In Hospital
ICD-10-CM	Z3800	Single Liveborn Infant, Delivered Vaginally
ICD-10-CM	Z3801	Single Liveborn Infant, Delivered By Cesarean
ICD-10-CM	Z381	Single Liveborn Infant, Born Outside Hospital
ICD-10-CM	Z382	Single Liveborn Infant, Unspecified As To Place Of Birth
ICD-10-CM	Z383	Twin Liveborn Infant, Born In Hospital
ICD-10-CM	Z3830	Twin Liveborn Infant, Delivered Vaginally
ICD-10-CM	Z3831	Twin Liveborn Infant, Delivered By Cesarean
ICD-10-CM	Z384	Twin Liveborn Infant, Born Outside Hospital
ICD-10-CM	Z385	Twin Liveborn Infant, Unspecified As To Place Of Birth
ICD-10-CM	Z386	Other Multiple Liveborn Infant, Born In Hospital
ICD-10-CM	Z3861	Triplet Liveborn Infant, Delivered Vaginally
ICD-10-CM	Z3862	Triplet Liveborn Infant, Delivered By Cesarean
ICD-10-CM	Z3863	Quadruplet Liveborn Infant, Delivered Vaginally
ICD-10-CM	Z3864	Quadruplet Liveborn Infant, Delivered By Cesarean
ICD-10-CM	Z3865	Quintuplet Liveborn Infant, Delivered Vaginally
ICD-10-CM	Z3866	Quintuplet Liveborn Infant, Delivered By Cesarean
ICD-10-CM	Z3868	Other Multiple Liveborn Infant, Delivered Vaginally
ICD-10-CM	Z3869	Other Multiple Liveborn Infant, Delivered By Cesarean
ICD-10-CM	Z387	Other Multiple Liveborn Infant, Born Outside Hospital
ICD-10-CM	Z388	Other Multiple Liveborn Infant, Unspecified As To Place Of Birth
ICD-9-CM	V30	Single Liveborn
ICD-9-CM	V300	Single Liveborn Born In Hospital
ICD-9-CM	V3000	Single Liveborn, Born In Hospital, Delivered Without Mention Of Cesarean Section
ICD-9-CM	V3001	Single Liveborn, Born In Hospital, Delivered By Cesarean Section
ICD-9-CM	V301	Single Liveborn, Born Before Admission To Hospital
ICD-9-CM	V31	Twin Birth Mate Liveborn
ICD-9-CM	V310	Twin Birth Mate Liveborn Born In Hospital
ICD-9-CM	V3100	Twin Birth, Mate Liveborn, Born In Hospital, Delivered Without Mention Of Cesarean Section

ICD-9-CM	V3101	Twin Birth, Mate Liveborn, Born In Hospital, Delivered By Cesarean Section
ICD-9-CM	V311	Twin Birth, Mate Liveborn, Born Before Admission To Hospital<ca>
ICD-9-CM	V32	Twin Birth Mate Stillborn
ICD-9-CM	V320	Twin Birth Mate Stillborn Born In Hospital
ICD-9-CM	V3200	Twin Birth, Mate Stillborn, Born In Hospital, Delivered Without Mention Of Cesarean Section
ICD-9-CM	V3201	Twin Birth, Mate Stillborn, Born In Hospital, Delivered By Cesarean Section<ca>
ICD-9-CM	V321	Twin Birth, Mate Stillborn, Born Before Admission To Hospital<ca>
ICD-9-CM	V33	Twin Birth Unspecified Whether Mate Liveborn Or Stillborn
ICD-9-CM	V330	Twin Birth Unspecified Whether Mate Liveborn Or Stillborn Born In Hospital
ICD-9-CM	V3300	Twin Birth, Unspecified Whether Mate Liveborn Or Stillborn, Born In Hospital, Delivered Without Mention Of Cesarean Section
ICD-9-CM	V3301	Twin Birth, Unspecified Whether Mate Liveborn Or Stillborn, Born In Hospital, Delivered By Cesarean Section
ICD-9-CM	V331	Twin Birth, Unspecified Whether Mate Liveborn Or Stillborn, Born Before Admission To Hospital
ICD-9-CM	V34	Other Multiple Birth (Three Or More) Mates All Liveborn
ICD-9-CM	V340	Other Multiple Birth (Three Or More) Mates All Liveborn Born In Hospital
ICD-9-CM	V3400	Other Multiple Birth (Three Or More), Mates All Liveborn, Born In Hospital, Delivered Without Mention Of Cesarean Section
ICD-9-CM	V3401	Other Multiple Birth (Three Or More), Mates All Liveborn, Born In Hospital, Delivered By Cesarean Section
ICD-9-CM	V341	Other Multiple Birth (Three Or More), Mates All Liveborn, Born Before Admission To Hospital
ICD-9-CM	V35	Other Multiple Birth (Three Or More) Mates All Stillborn
ICD-9-CM	V350	Other Multiple Birth (Three Or More), Mates All Still Born, Born In Hospital
ICD-9-CM	V3500	Other Multiple Birth (Three Or More), Mates All Still Born, Born In Hospital, Delivered Without Mention Of Cesarean Section
ICD-9-CM	V3501	Other Multiple Birth (Three Or More), Mates All Still Born, Born In Hospital, Delivered By Cesarean Section
ICD-9-CM	V351	Other Multiple Birth (Three Or More), Mates All Stillborn, Born Before Admission To Hospital
ICD-9-CM	V36	Other Multiple Birth (Three Or More) Mates Liveborn And Stillborn
ICD-9-CM	V360	Other Multiple Birth (Three Or More) Mates Liveborn And Stillborn Born In Hospital
ICD-9-CM	V3600	Other Multiple Birth (Three Or More), Mates Liveborn And Stillborn, Born In Hospital, Delivered Without Mention Of Cesarean Section
ICD-9-CM	V3601	Other Multiple Birth (Three Or More), Mates Liveborn And Stillborn, Born In Hospital, Delivered Without Mention Of Cesarean Section
ICD-9-CM	V361	Other Multiple Birth (Three Or More), Mates Liveborn And Stillborn, Born Before Admission To Hospital
ICD-9-CM	V37	Other Multiple Birth (Three Or More) Unspecified Whether Mates Liveborn Or Stillborn
ICD-9-CM	V370	Other Multiple Birth (Three Or More) Unspecified Whether Mates Liveborn Or Stillborn Born In Hospital
ICD-9-CM	V3700	Other Multiple Birth (Three Or More), Unspecified Whether Mates Liveborn Or Stillborn, Born In Hospital, Delivered Without Mention Of Cesarean Section
ICD-9-CM	V3701	Other Multiple Birth (Three Or More), Unspecified Whether Mates Liveborn Or Stillborn, Born In Hospital, Delivered By Cesarean Section
ICD-9-CM	V371	Other Multiple Birth (Three Or More), Unspecified Whether Mates Liveborn Or Stillborn, Born Before Admission To Hospital
ICD-9-CM	V39	Liveborn Unspecified Whether Single Twin Or Multiple
ICD-9-CM	V390	Liveborn Unspecified Whether Single Twin Or Multiple Born In Hospital

ICD-9-CM	V3900	Liveborn, Unspecified Whether Single, Twin Or Multiple, Born In Hospital, Delivered Without Mention Of Cesarean Section
ICD-9-CM	V3901	Liveborn, Unspecified Whether Single, Twin Or Multiple, Born In Hospital, Delivered By Cesarean Section
ICD-9-CM	V391	Liveborn, Unspecified Whether Single, Twin Or Multiple, Born Before Admission To Hospital

Table 5 shows our list of twin or multiple birth codes. Any newborn with a claim containing one of these codes was excluded from our matching process.

Table 5: Medicaid Newborn Codes for Twins

Code	Code Type	Description
Z372	ICD-10-CM	Twins, Both Liveborn
Z375	ICD-10-CM	Other Multiple Births, All Liveborn
Z3750	ICD-10-CM	Multiple Births, Unspecified, All Liveborn
Z3751	ICD-10-CM	Triplets, All Liveborn
Z3752	ICD-10-CM	Quadruplets, All Liveborn
Z3753	ICD-10-CM	Quintuplets, All Liveborn
Z3754	ICD-10-CM	Sextuplets, All Liveborn
Z3759	ICD-10-CM	Other Multiple Births, All Liveborn
Z376	ICD-10-CM	Other Multiple Births, Some Liveborn
Z3760	ICD-10-CM	Multiple Births, Unspecified, Some Liveborn
Z3761	ICD-10-CM	Triplets, Some Liveborn
Z3762	ICD-10-CM	Quadruplets, Some Liveborn
Z3763	ICD-10-CM	Quintuplets, Some Liveborn
Z3764	ICD-10-CM	Sextuplets, Some Liveborn
Z3769	ICD-10-CM	Other Multiple Births, Some Liveborn
Z377	ICD-10-CM	Other Multiple Births, All Stillborn
Z383	ICD-10-CM	Twin Liveborn Infant, Born In Hospital
Z3830	ICD-10-CM	Twin Liveborn Infant, Delivered Vaginally
Z3831	ICD-10-CM	Twin Liveborn Infant, Delivered By Cesarean
Z384	ICD-10-CM	Twin Liveborn Infant, Born Outside Hospital
Z385	ICD-10-CM	Twin Liveborn Infant, Unspecified As To Place Of Birth
Z386	ICD-10-CM	Other Multiple Liveborn Infant, Born In Hospital
Z3861	ICD-10-CM	Triplet Liveborn Infant, Delivered Vaginally
Z3862	ICD-10-CM	Triplet Liveborn Infant, Delivered By Cesarean
Z3863	ICD-10-CM	Quadruplet Liveborn Infant, Delivered Vaginally
Z3864	ICD-10-CM	Quadruplet Liveborn Infant, Delivered By Cesarean
Z3865	ICD-10-CM	Quintuplet Liveborn Infant, Delivered Vaginally
Z3866	ICD-10-CM	Quintuplet Liveborn Infant, Delivered By Cesarean
Z3868	ICD-10-CM	Other Multiple Liveborn Infant, Delivered Vaginally
Z3869	ICD-10-CM	Other Multiple Liveborn Infant, Delivered By Cesarean
Z387	ICD-10-CM	Other Multiple Liveborn Infant, Born Outside Hospital
Z388	ICD-10-CM	Other Multiple Liveborn Infant, Unspecified As To Place Of Birth
V31	ICD-9-CM	Twin Birth Mate Liveborn
V310	ICD-9-CM	Twin Birth Mate Liveborn Born In Hospital
V3100	ICD-9-CM	Twin Birth, Mate Liveborn, Born In Hospital, Delivered Without Mention Of Cesarean Section
V3101	ICD-9-CM	Twin Birth, Mate Liveborn, Born In Hospital, Delivered By Cesarean Section
V311	ICD-9-CM	Twin Birth, Mate Liveborn, Born Before Admission To Hospital
V312	ICD-9-CM	Twin Birth, Mate Liveborn, Born Outside Hospital And Not Hospitalized
V33	ICD-9-CM	Twin Birth Unspecified Whether Mate Liveborn Or Stillborn
V330	ICD-9-CM	Twin Birth Unspecified Whether Mate Liveborn Or Stillborn Born In Hospital
V3300	ICD-9-CM	Twin Birth, Unspecified Whether Mate Liveborn Or Stillborn, Born In Hospital, Delivered Without Mention Of Cesarean Section
V3301	ICD-9-CM	Twin Birth, Unspecified Whether Mate Liveborn Or Stillborn, Born In Hospital, Delivered By Cesarean Section
V331	ICD-9-CM	Twin Birth, Unspecified Whether Mate Liveborn Or Stillborn, Born Before Admission To Hospital

V332	ICD-9-CM	Twin Birth, Unspecified Whether Mate Liveborn Or Stillborn, Born Outside Hospital And Not Hospitalized
V34	ICD-9-CM	Other Multiple Birth (Three Or More) Mates All Liveborn
V340	ICD-9-CM	Other Multiple Birth (Three Or More) Mates All Liveborn Born In Hospital
V3400	ICD-9-CM	Other Multiple Birth (Three Or More), Mates All Liveborn, Born In Hospital, Delivered Without Mention Of Cesarean Section
V3401	ICD-9-CM	Other Multiple Birth (Three Or More), Mates All Liveborn, Born In Hospital, Delivered By Cesarean Section
V341	ICD-9-CM	Other Multiple Birth (Three Or More), Mates All Liveborn, Born Before Admission To Hospital
V342	ICD-9-CM	Other Multiple Birth (Three Or More), Mates All Liveborn, Born Outside Hospital And Not Hospitalized
V36	ICD-9-CM	Other Multiple Birth (Three Or More) Mates Liveborn And Stillborn
V360	ICD-9-CM	Other Multiple Birth (Three Or More) Mates Liveborn And Stillborn Born In Hospital
V3600	ICD-9-CM	Other Multiple Birth (Three Or More), Mates Liveborn And Stillborn, Born In Hospital, Delivered Without Mention Of Cesarean Section
V3601	ICD-9-CM	Other Multiple Birth (Three Or More), Mates Liveborn And Stillborn, Born In Hospital, Delivered Without Mention Of Cesarean Section
V361	ICD-9-CM	Other Multiple Birth (Three Or More), Mates Liveborn And Stillborn, Born Before Admission To Hospital
V362	ICD-9-CM	Other Multiple Birth (Three Or More), Mates Liveborn And Stillborn, Born Outside Hospital And Not Hospitalized
V37	ICD-9-CM	Other Multiple Birth (Three Or More) Unspecified Whether Mates Liveborn Or Stillborn
V370	ICD-9-CM	Other Multiple Birth (Three Or More) Unspecified Whether Mates Liveborn Or Stillborn Born In Hospital
V3700	ICD-9-CM	Other Multiple Birth (Three Or More), Unspecified Whether Mates Liveborn Or Stillborn, Born In Hospital, Delivered Without Mention Of Cesarean Section
V3701	ICD-9-CM	Other Multiple Birth (Three Or More), Unspecified Whether Mates Liveborn Or Stillborn, Born In Hospital, Delivered By Cesarean Section
V371	ICD-9-CM	Other Multiple Birth (Three Or More), Unspecified Whether Mates Liveborn Or Stillborn, Born Before Admission To Hospital
V372	ICD-9-CM	Other Multiple Birth (Three Or More), Unspecified Whether Mates Liveborn Or Stillborn, Born Outside Of Hospital

When using birth related claims to identify newborns, we find fewer newborns than we expect to compared to our NVSS benchmark. Care for newborns can be billed under their mother's ID number. These claims are excluded from our analysis on the basis of "beneficiaries" being over 1 year old. While we are only identifying 0.44% fewer births than we expect nationally, in the populous state of California, we are identifying 83.68% fewer births than we expect when only relying on claims for newborn care.

In order to identify newborns nationwide, we identify state/years in which we suspect that care for newborns was frequently billed under the mothers' IDs and use enrollment records to identify these newborns. Specifically, we identify state/year combinations in which we are under-identifying newborns, based on our NVSS newborns data, by at least 5%. This list of state/year combinations can be found in Table 6 below. For each of these state/year combinations, we find all beneficiaries born in the state/year who do not have any claims for newborn care, but who are enrolled in Medicaid anytime before their birth, on their birth date, or on the day after their birth date. We include beneficiaries enrolled before their date of birth because some states document the start date of a beneficiary's enrollment as the first day of the month or the year in which they were born. We exclude young beneficiaries enrolled more than one day after birth in order to minimize the inclusion of infants who were not born to Medicaid beneficiaries.

For newborns, we identify without birth claims, it is more difficult to identify twins. For these newborns, we nonetheless use the newborn twin or multiple birth codes from Table 5 to determine if any claims up to a month after a newborn's birth would indicate multiple birth. If any single claim includes one of these codes, then that newborn is designated as a multiple birth and is subsequently excluded from the matching process.

Table 6: State/Years with Birth Claims for less than 95% Expected Number of Newborns

State	Years
California	2011-2019
Colorado	2011-2019
District of Columbia	2011-2017, 2019
Idaho	2011
Illinois	2011-2013
Louisiana	2011-2012
Maine	2011
Massachusetts	2011
Mississippi	2015-2016
Missouri	2015
Nevada	2011-2014
New Hampshire	2017-2019
New Jersey	2011-2019
Pennsylvania	2011-2012
Rhode Island	2011, 2013-2019
Tennessee	2011-2019
Vermont	2011, 2013-2016
Virginia	2011-2015
Washington	2011-2019
West Virginia	2011-2016, 2018-2019

3.2 Total Newborns by State and Year

Using enrollment records to supplement our claims-based identification process allows us to identify newborns in all 50 states. Significant improvements can be seen in comparing Figures 3.1 and 3.2 for California, Colorado, and West Virginia. However, this method ultimately identifies more newborns than the number of newborns covered by Medicaid reported by NVSS. Overall, when including newborns enrolled at birth, we have 17.85% more newborns than the reported number covered under Medicaid in NVSS.

Figure 3.1: Newborns by State and Year - Just Birth Claims

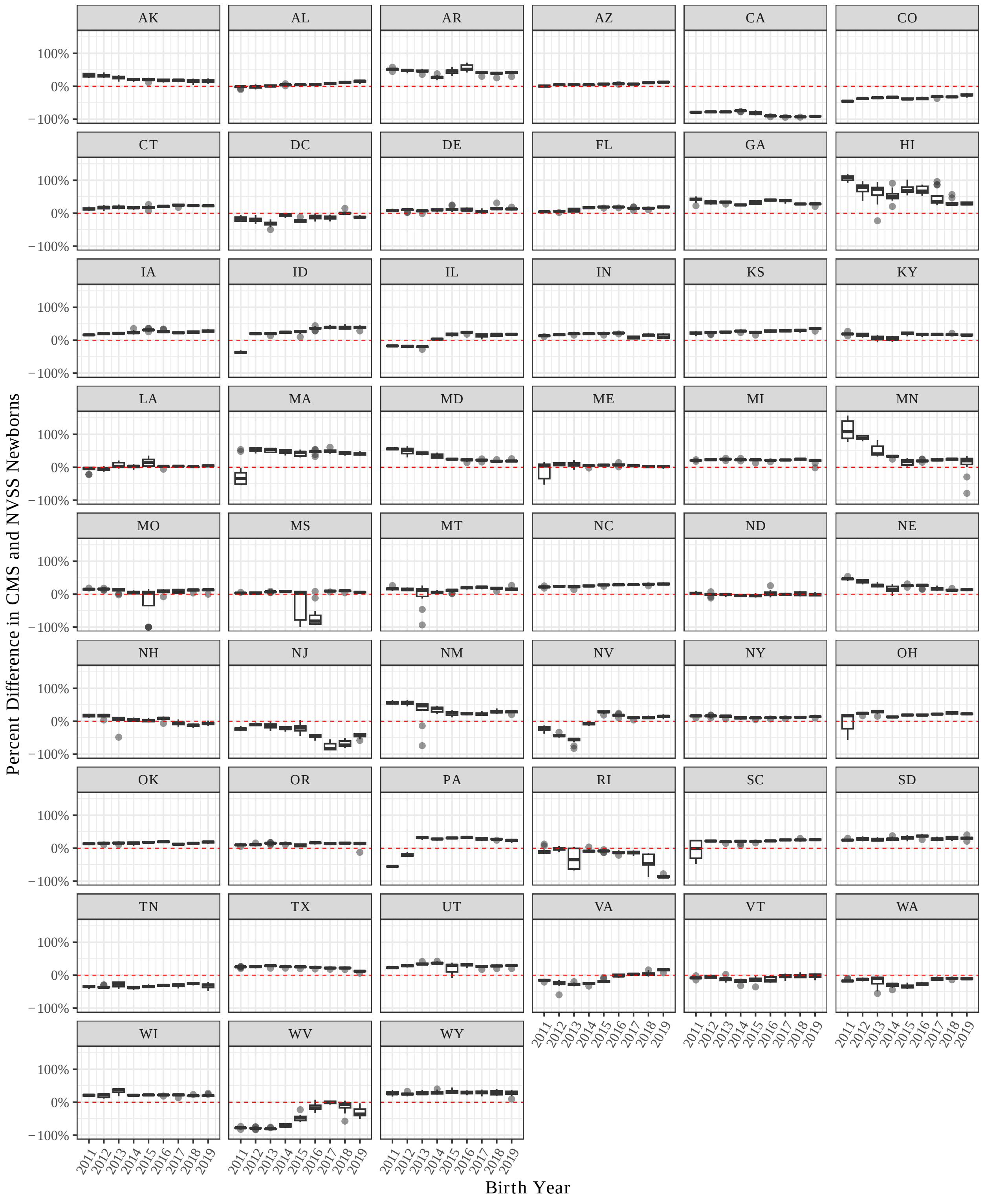

Figure 3.2: Newborns by State and Year - Total

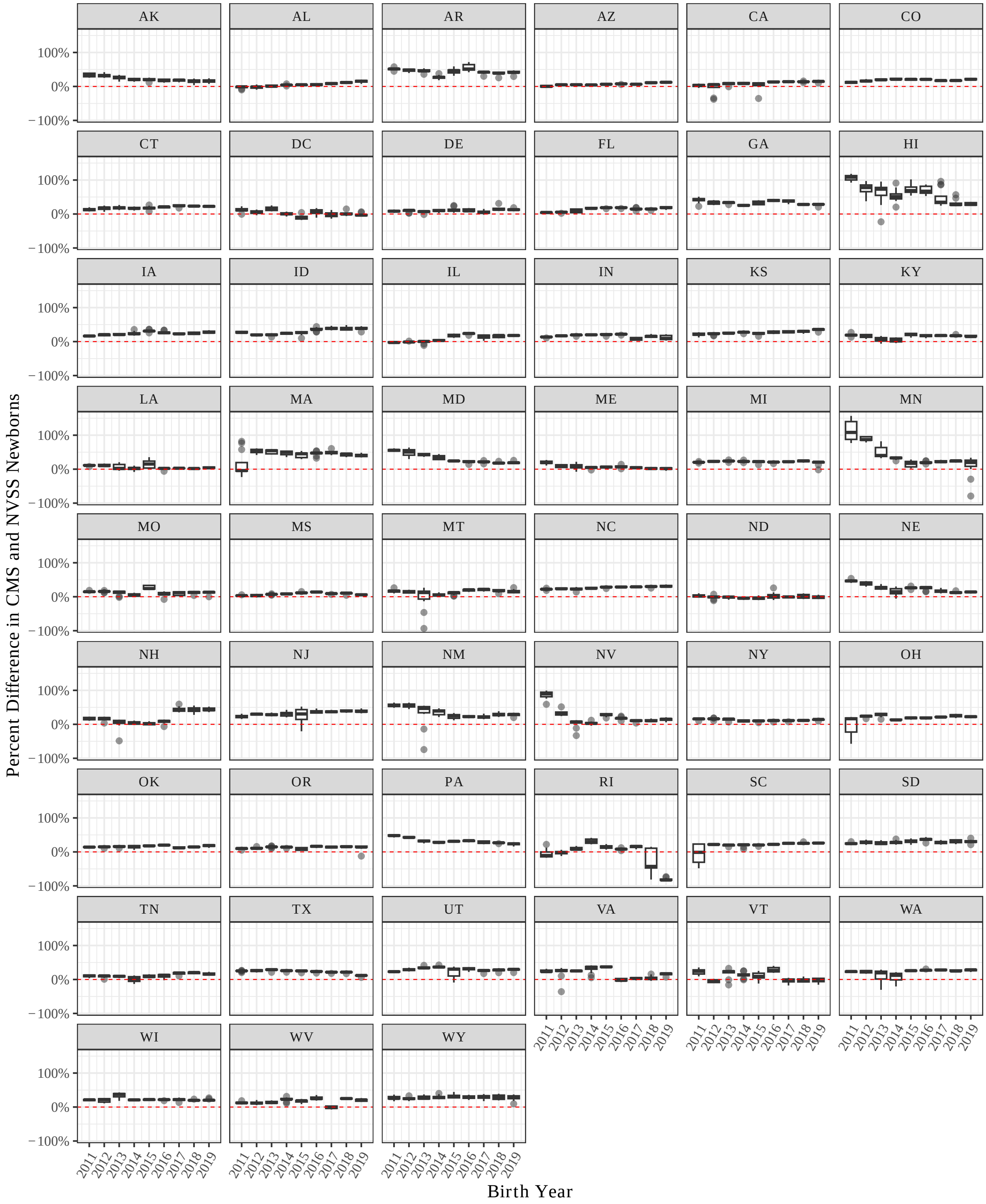

3.3 Identifying Outliers by State/Year

Figure 3.3 shows a distribution of the percent difference between the number of our identified newborns and the number of NVSS newborns by state and year. The dotted lines indicate the 5th percentile and 95th percentile of these differences. Because our method is designed identify at least as many newborns as seen our NVSS newborns benchmark, our 5th percentile is a -1.14% difference and our 95th percentile is a 46.41% difference.

Figure 3.3: Percent Difference of CMS and NVSS Newborns by State/Year

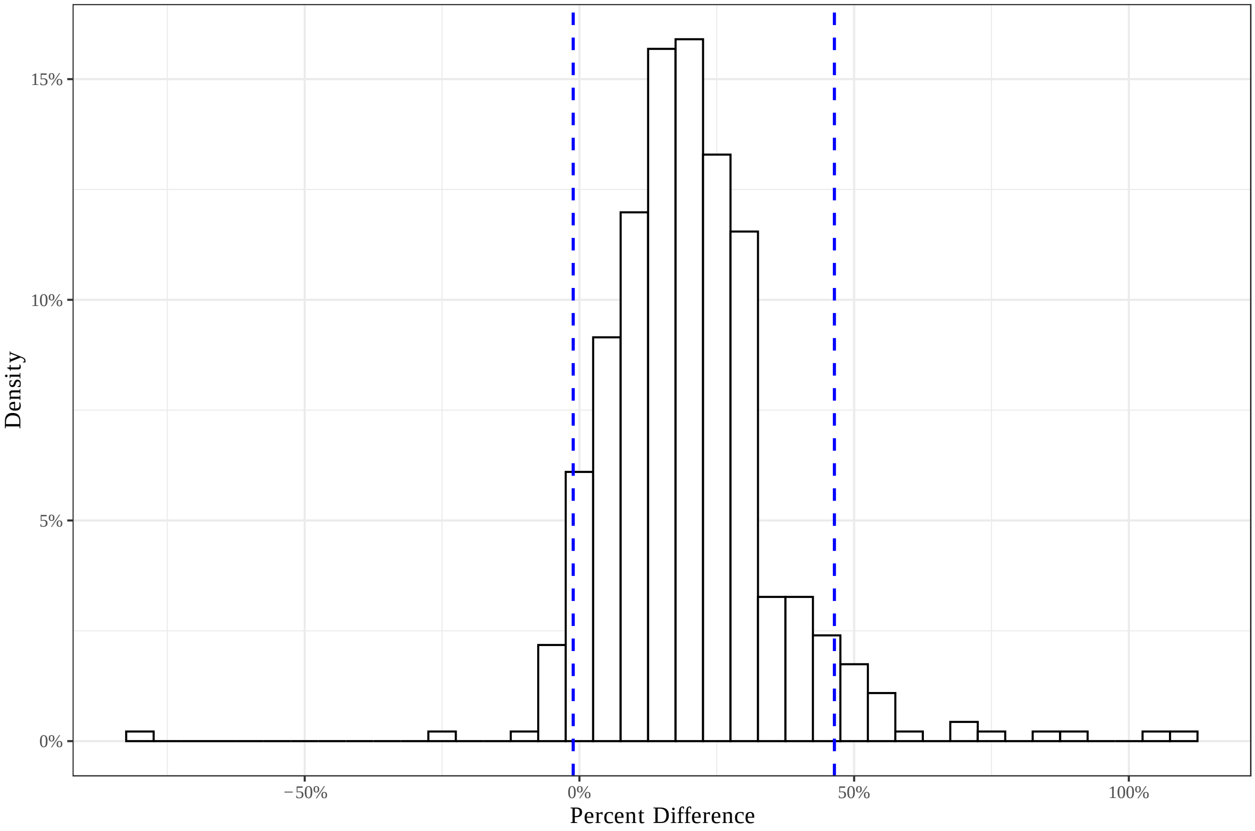

Figure 3.4 is a plot of percent differences by state and year, indicating state/year combinations that do not fall in the middle 90% of the distribution of percent differences. Similarly to Figure 2.5, this figure highlights our overestimation of newborns in certain states across consecutive years. In states like Massachusetts, Hawaii, and Minnesota, we are over-identifying newborns across consecutive years, which could indicate a problem in our newborn identification method. However, like with deliveries, it could specify a change or problem with reporting of newborns in those states for certain periods of time.

Figure 3.4: Percent Difference of CMS and NVSS Newborns by State/Year - Outliers

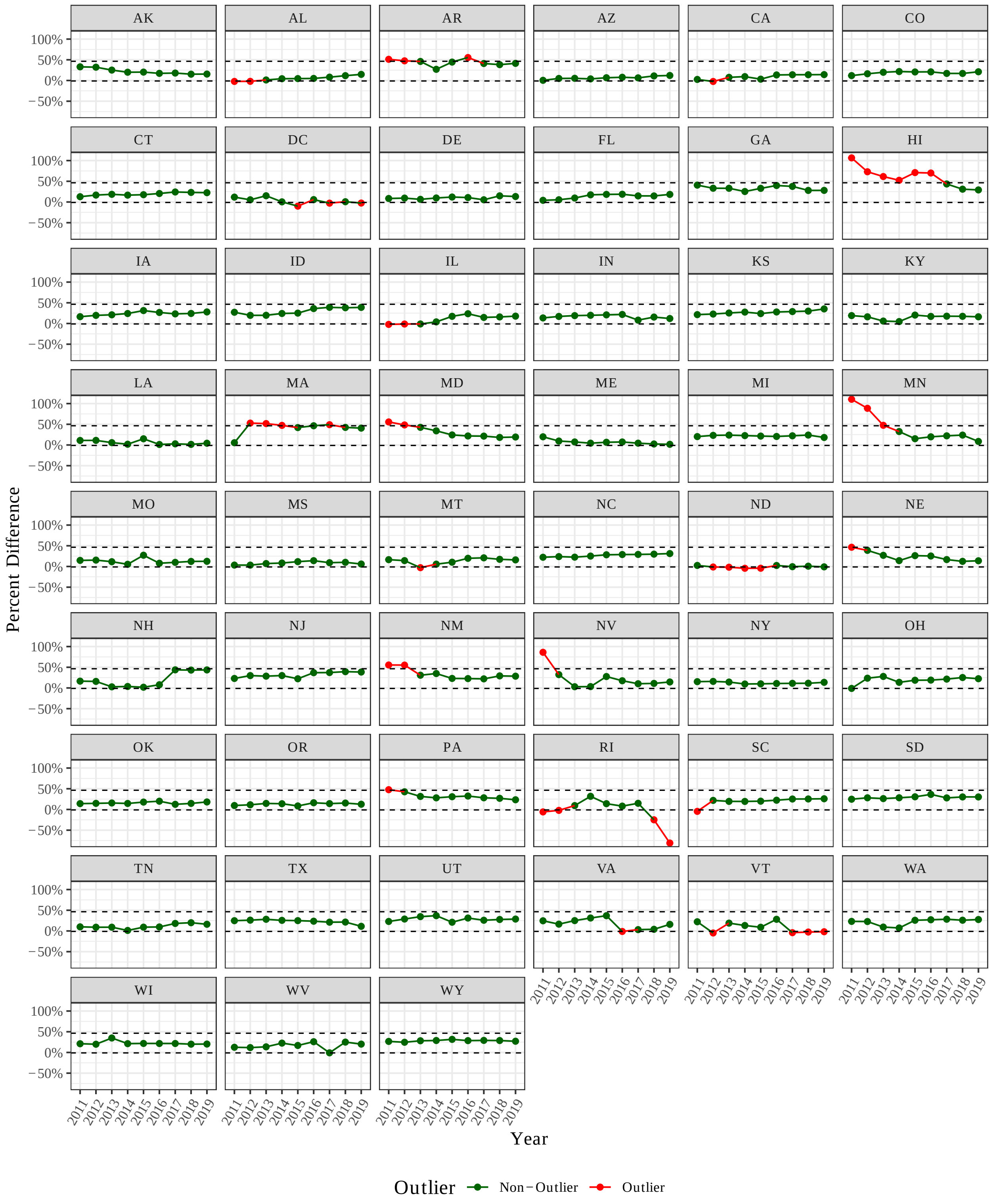

4 Data Cleaning

4.1 Delivery Date Window

Once we have finished identifying our deliveries using claims for delivery-related care and our clustering algorithm, we identify a plausible delivery window for each delivery. We create a delivery procedure code flag using all of the 14 CPT codes from our 96 delivery codes. If there is a delivery procedure code flag in a given cluster, then the service begin date and service end date for that claim are used to set the beginning and end of the delivery window for that cluster. If there is more than one delivery procedure code flag in a given cluster, then the earliest service begin date and latest service end date for those claims are used as the beginning and end of the delivery window. If there is no delivery procedure code flag for a given cluster, then the earliest service begin date for all claims in the cluster and the latest service end date are the beginning and end of the delivery window.

Figure 4.1 shows the distribution of the length of our delivery windows in days. As we can see from this figure, the overwhelming majority of our delivery windows span zero days between the delivery begin date and delivery end date. In other words, for 81.27% of our identified deliveries, we have narrowed down the delivery date to the day. For the rest of our deliveries, we have a range of days in which the actual delivery date would fall.

Figure 4.1: Distribution of Length of Delivery Window

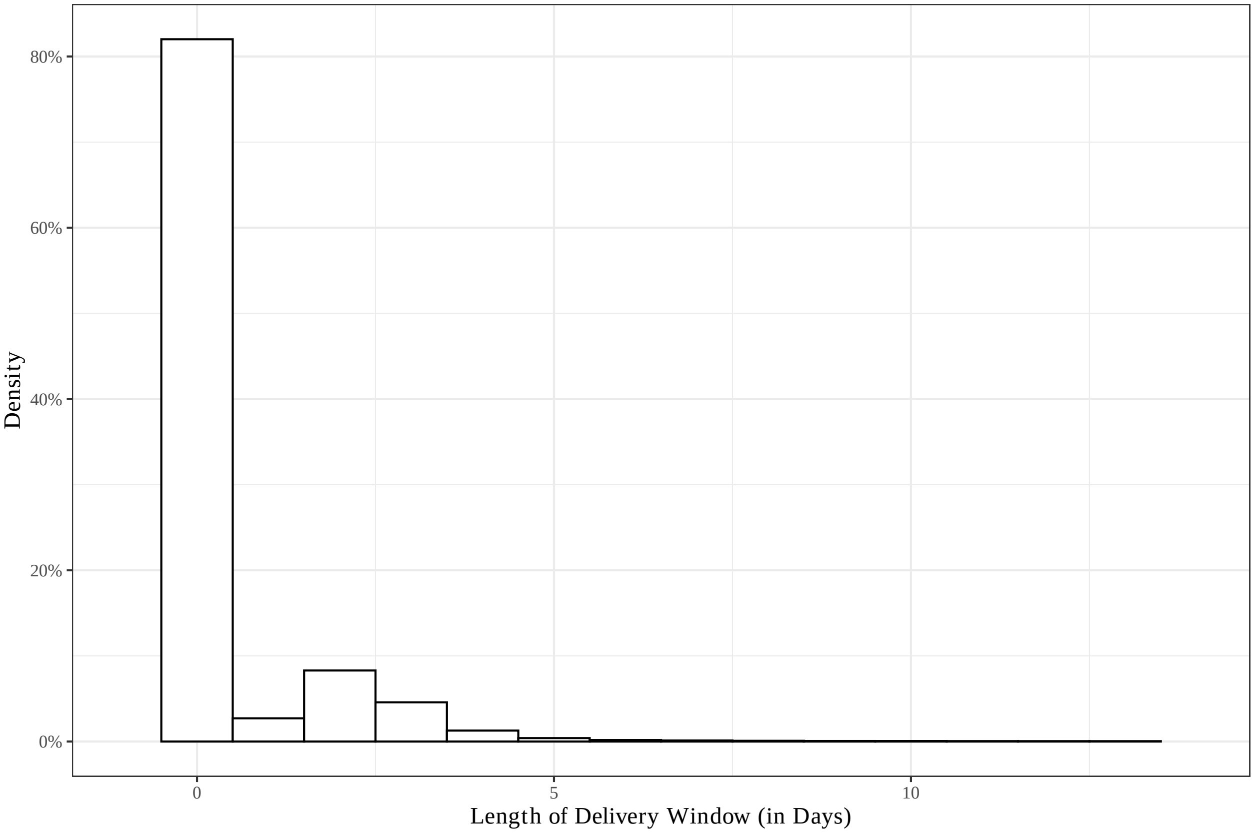

Before matching, we filter out any deliveries whose delivery window is longer than 7 days. This filters out 213,101 deliveries, which is 1.32% of our total universe of deliveries up to this point.

4.2 Birth Date

Unlike the rest of the demographic variables in our data, we merge demographic birth date (the date recorded in enrollment records) into claims data prior to identifying newborns and deliveries. We do this in order to filter possible newborns and deliveries by age. This is critical because claims for infants somewhat frequently include the demographic information (including date of birth) of one of the parents instead of that of the child.

In order to merge birth dates from our demographic data into our claims data, we collect the birth dates of each beneficiary from the demographic files for every year from 2011 to 2019. We use this to fill any missingness across years for a beneficiary. If a beneficiary has more than one demographic birth date, we leave their demographic birth date as missing. Any beneficiaries with missing demographic birth dates are excluded entirely from our universe of newborns or deliveries because of the age filter.

Using this method, 99.52% of beneficiaries have a demographic birth date across all years and claims types.

4.3 Residential Zip Code

In order to merge residential zip codes from our demographic data to our newborns and deliveries data, we collect residential zip codes for each of our beneficiaries from the demographic files for every year from 2011 to 2019. We then fill any missing values across years for each beneficiary. If a beneficiary has a missing residential zip code for a given year, we assume the most recently reported residential zip code until a new one appears in our demographic data. We then record any residential zip codes that are 99999 or start with 000 as missing. Finally, we merge residential zip codes into our newborn and delivery data by beneficiary (MSIS ID and state) and year (year of birth or year at the start of a delivery window).

Table 7 presents information on the missingness of residential zip codes for our newborns and deliveries before matching.

Table 7: Residential Zip Code Missingness

Deliveries or Newborns	Not Missing	Missing
Deliveries	99.2%	0.8%
Newborns	92.4%	7.6%

4.4 Facility Zip Code

In order to determine the zip code of the facility in which a delivery took place and in which an infant was born, we use information from our claims data rather than our demographic data.

For newborns, this means that we only identify facility zip codes for beneficiaries for whom we have birth claims. For any newborns that are identified without birth claims, we do not include a facility zip code.

For deliveries and newborns with birth related claims, we extract every NPI (with the exception of referring physician NPIs) from each of their delivery/birth claims and find the facility zip code associated with each NPI in NPPES registry data from the year of the claim. If multiple different zip codes are associated with NPIs listed as care providers on the claims, the most common zip code is used as our estimate for facility location. If no zip code appears more frequently than others, we do not estimate a facility zip code for the delivery or birth.

Lastly, we recode any facility zip codes that are 99999 or start with 000 as missing.

Table 8 presents information on the missingness of facility zip codes for our newborns and deliveries before matching.

Table 8: Facility Zip Code Missingness

Deliveries or Newborns	Not Missing	Missing
Newborns	69.4%	30.6%
Deliveries	78.8%	21.2%

4.5 Race and Ethnicity

Merging our race and ethnicity information from our demographic data into our newborns and deliveries data is very similar to the process we use to merge our residential zip code information. We collect race/ethnicity for each of our beneficiaries from the demographic files for every year from 2011 to 2019. We then fill any missing values across years for each beneficiary. If a beneficiary has missing race/ethnicity information for a given year, we use the most recently reported race/ethnicity information. If a beneficiary has a race/ethnicity code that corresponds to "Unknown" or "Multiracial," we set that beneficiary's race/ethnicity code missing while matching. This allows us to avoid concluding that two beneficiaries with unknown race share a racial identify, and to avoid concluding that a multiracial beneficiary has a different race than anyone with a single racial identity. For MAX data, this corresponds to the codes 8 and 10. For TAF data, this is code 6. If there is more than one race/ethnicity for a given beneficiary across years, that beneficiary is given a missing race/ethnicity for all years.

Table 9 presents information on the missingness of race/ethnicity for our newborns and deliveries before matching.

Table 9: Race/Ethnicity Missingness

Deliveries or Newborns	Not Missing	Missing
Newborns	69%	31%
Deliveries	90%	10%

4.6 Case Number

Case number is merged into our newborns and deliveries data from our demographic data in the exact same way that residential zip code is. One additional precaution we take with case number is to remove any case numbers seen in our demographic data 10 times or more. These case numbers are made to be missing in our newborns and deliveries data before matching because we do not believe that any case numbers assigned to 10 or more people were likely to have been assigned at the household level.

In our universe of pre-matched deliveries and newborns, these "common" case numbers appear for 2.91% of our deliveries and 2.1% of our newborns.

Table 10 presents information on the missingness of case number for our newborns and deliveries before matching. Note than many of the observed case numbers here are assigned at the individual level and are not ultimately useful for identifying family or household units.

Table 10: Case Number Missingness

Deliveries or Newborns	Not Missing	Missing
Deliveries	96.9%	3.1%
Newborns	90.6%	9.4%

4.7 Overall Missingness

Figure 4.2 is a plot of the missingness of our demographic variables by year for newborns and deliveries. The gray lines indicate the trend in missingness for individual states, while the thick black lines indicate the trend

in missingness nationwide. Except for newborn missingness for race/ethnicity, missingness generally appears to go down or stay the same across years. Delivery facility zip code is more stable and more often available from TAF data than MAX data, but otherwise we do not observe unusual changes in data availability around the time of the MAX to TAF transition.

Figure 4.2: Missingness of Demographic Variables - Newborns and Deliveries by State and Year

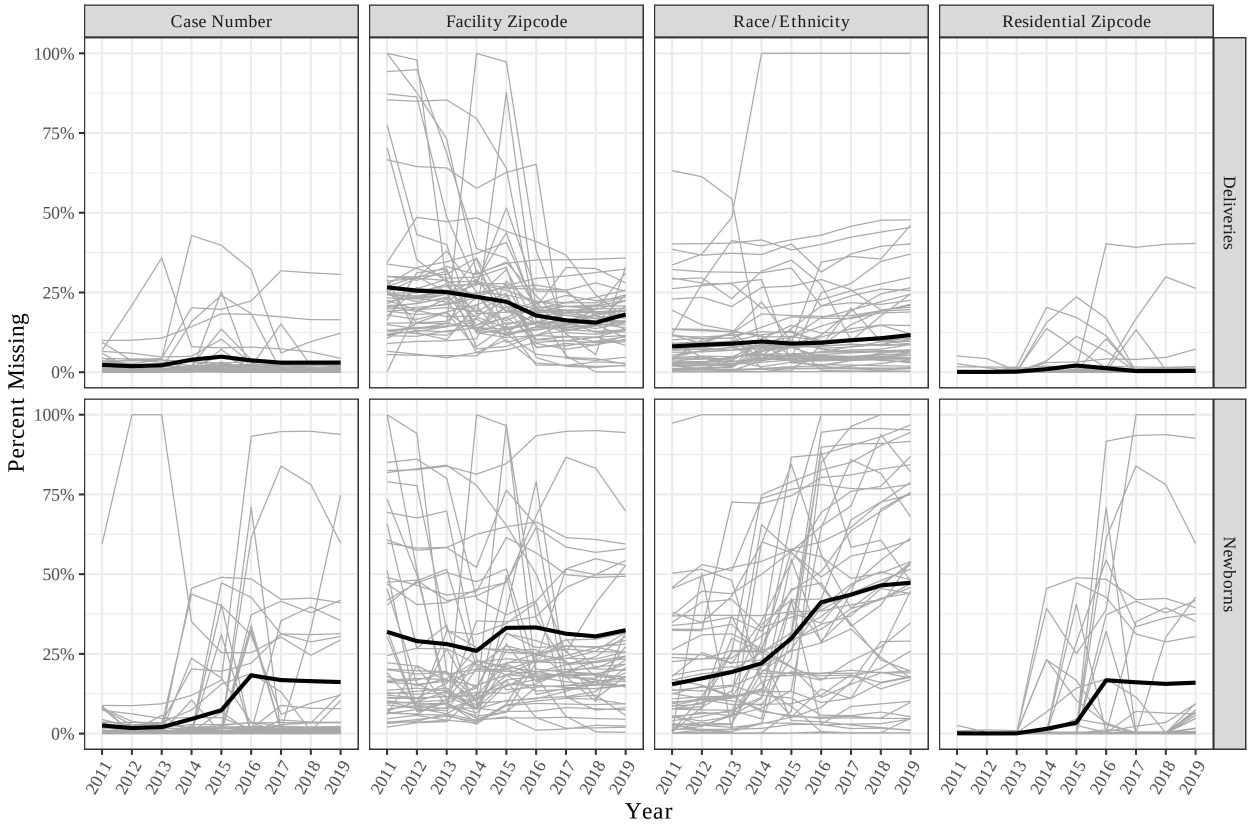

5 Matching

5.1 Description of Matching

Our matching revolves around matching on state, newborn date of birth, and delivery date window, as well as 4 different demographic variables: residential zip code, facility zip code, race/ethnicity, and case number. We note that while we do rely on indirect identifiers and protected health information, no beneficiaries were reidentified in the course of our analysis. Working in a secure computing environment, we implemented 6 rounds of matching. The first 3 rounds have 8 steps each and the last 3 rounds have 6 steps each.

For rounds 1-3 of our matching algorithm, different iterations of variable combinations are used to match, with case number and state always being included. The first step of these 3 rounds matches on all possible variables. The second step matches on state, case number, facility zip code, and residential zip code, meaning that if race/ethnicity is missing or mismatching, it can still lead to a linked dyad. The third step matches on state, case number, residential zip code, and race/ethnicity. The fourth step matches on state, case number, facility zip code, and race/ethnicity. The fifth step matches on state, case number, and residential zip code. The sixth step matches on state, case number, and facility zip code. The seventh step matches on state, case number, and race/ethnicity. Finally, the eighth step matches on state and case number only.

Round 1 is predicated on a newborn's date of birth falling withing the bounds of a delivery window, while matching all of the different iterations of demographic variable combinations as described above. Round 2 increases the original delivery window by a day on each side. Round 3 increases the original delivery window by two days on each side.

For rounds 4-6 of our matching algorithm, different iterations of variable combinations are used to match, with only state being included. Case number is completely excluded from the matching in these last 3 rounds, as case number is not always assigned at the household level. Texas, for instance, is an example of a state that does not typically give family members the same case numbers. This is one of the reasons why our matching rate for Texas through the first 3 rounds is particularly low compared to other states. The first step of these last 3 rounds matches on all possible variables. The second step matches on state, facility zip code, and residential zip code. The third step matches on state, residential zip code, and race/ethnicity. The fourth step matches on state, facility zip code, and race/ethnicity. The fifth step matches on state and residential zip code. Finally the sixth step matches on state and facility zip code.

Similarly to the first three rounds, round 4 is predicated on a newborn's date of birth falling withing the bounds of a delivery window, while matching all of the different iterations of demographic variable combinations as described above, excluding case number. Round 5 increases the original delivery window by a day on each side. Round 6 increases the original delivery window by two days on each side.

Table 11 is a summary of how many matched dyads share demographic characteristics. This includes when demographic characteristics between matched dyads are both missing.

Table 11: Matched Dyads that Share Demographic Characteristics

Demographic Variables	Matching Percentage	Conflicting Percentage	Both Missing Percentage	One Missing Percentage
Residential Zip Code	92.90%	6.69%	0.07%	0.34%
Race/Ethnicity	65.86%	7.40%	6.77%	19.98%
Case Number	65.82%	31.76%	1.45%	0.97%
Facility Zip Code	42.48%	21.94%	7.10%	28.48%

5.2 Matching Rates by State

Table 12 describes our matching rates and total number of matched dyads through round 3 and round 6 for both newborns and deliveries by state. The reason we care about rounds 3 and 6 for these diagnostics is

Figure 5.1: Criteria for Linking Newborns and Deliveries

Phase	Step	Date	State	Case ID Number	Facility Zipcode (from NPI)	Residential Zipcode	Race/Ethnicity	Matches	Cumulative Matches	% Deliveries Matched	% Newborns Matched
1	1	DOB between beginning of delivery window and end of delivery window	X	X	X	X	X	1,822,171	1,822,171	11.6%	10.5%
	2		X	X	X	X		905,394	2,727,565	17.3%	15.8%
	3		X	X		X	X	2,936,768	5,664,333	36.0%	32.7%
	4		X	X	X		X	101,817	5,766,150	36.7%	33.3%
	5		X	X		X		1,355,066	7,121,216	45.3%	41.1%
	6		X	X	X			53,690	7,174,906	45.6%	41.5%
	7		X	X			X	165,232	7,340,138	46.7%	42.4%
	8		X	X				92,264	7,432,402	47.3%	42.9%
2	9	DOB between beginningof delivery window -1 and end of delivery window +1	X	X	X	X	X	52,333	7,484,735	47.6%	43.2%
	10		X	X	X	X		24,481	7,509,216	47.7%	43.4%
	11		X	X		X	X	96,653	7,605,869	48.4%	43.9%
	12		X	X	X		X	2,867	7,608,736	48.4%	44.0%
	13		X	X		X		42,946	7,651,682	48.6%	44.2%
	14		X	X	X			1,564	7,653,246	48.7%	44.2%
	15		X	X			X	5,643	7,658,889	48.7%	44.3%
	16		X	X				2,971	7,661,860	48.7%	44.3%
3	17	DOB between beginningof delivery window -2 and end of delivery window +2	X	X	X	X	X	5,645	7,667,505	48.7%	44.3%
	18		X	X	X	X		2,844	7,670,349	48.8%	44.3%
	19		X	X		X	X	11,873	7,682,222	48.8%	44.4%
	20		X	X	X		X	326	7,682,548	48.8%	44.4%
	21		X	X		X		5,432	7,687,980	48.9%	44.4%
	22		X	X	X			150	7,688,130	48.9%	44.4%
	23		X	X			X	705	7,688,835	48.9%	44.4%
	24		X	X				376	7,689,211	48.9%	44.4%
4	25	DOB between beginningof delivery window and end of delivery window	X		X	X	X	941,558	8,630,769	54.9%	49.9%
	26		X		X	X		540,439	9,171,208	58.3%	53.0%
	27		X			X	X	1,112,504	10,283,712	65.4%	59.4%
	28		X		X		X	175,121	10,458,833	66.5%	60.4%
	29		X			X		625,144	11,083,977	70.5%	64.0%
	30		X		X			106,718	11,190,695	71.1%	64.7%
5	31	DOB between beginningof delivery window -1 and end of delivery window +1	X		X	X	X	34,085	11,224,780	71.4%	64.9%
	32		X		X	X		35,755	11,260,535	71.6%	65.1%
	33		X			X	X	94,157	11,354,692	72.2%	65.6%
	34		X		X		X	44,873	11,399,565	72.5%	65.9%
	35		X			X		89,991	11,489,556	73.0%	66.4%
	36		X		X			36,314	11,525,870	73.3%	66.6%
6	37	DOB between beginningof delivery window -2 and end of delivery window +2	X		X	X	X	12,405	11,538,275	73.4%	66.7%
	38		X		X	X		16,052	11,554,327	73.5%	66.8%
	39		X			X	X	44,066	11,598,393	73.7%	67.0%
	40		X		X		X	21,910	11,620,303	73.9%	67.1%
	41		X			X		46,285	11,666,588	74.2%	67.4%
	42		X		X			17,751	11,684,339	74.3%	67.5%

because we match the overwhelming majority of our dyads in these two rounds, both nationwide and across all states. Through 3 rounds, we are matching 44.4% of our universe of newborns nationwide. Through 6 rounds, we are matching 67.5% of our universe of newborns nationwide.

For states like Connecticut, New Jersey, and Texas, we are starkly under-identifying matches through the first 3 rounds, leading to lower than average match rates through all 6 rounds for these states. This is because these states do not consistently use case number as a household ID. This makes it very difficult for us to match newborns and mothers using case number, which is the basis of the first 3 rounds of our matching algorithm. For the same reason, North Carolina has a low newborn match rate through 3 rounds, but it does not appear to affect the final newborn match rate as much as it does for these other 3 states. Conversely, we match the overwhelming majority of mothers and newborns for states like Delaware, Indiana, and New Hampshire in the first 3 rounds of our algorithm. Among other things, this underscores the usefulness of case number as a household ID in these states.

Table 12: Matching Diagnostics by State

State	Through Round 3			Through Round 6		
	Matched Dyads	Delivery Match %	Newborn Match %	Matched Dyads	Delivery Match %	Newborn Match %
Total	7,689,211	48.9%	44.4%	11,684,339	74.3%	67.5%
Alabama	183,606	76.0%	66.3%	205,457	85.0%	74.2%
Alaska	30,263	74.9%	70.8%	36,854	91.2%	86.3%
Arizona	311,348	77.5%	75.3%	369,917	92.1%	89.4%
Arkansas	8,427	6.3%	4.1%	86,114	64.6%	41.6%
California	628,064	33.1%	30.6%	860,455	45.3%	41.9%
Colorado	137,891	58.8%	53.4%	156,013	66.5%	60.4%
Connecticut	-	0.0%	0.0%	86,168	64.6%	61.3%
Delaware	40,831	86.1%	84.3%	43,803	92.4%	90.5%
District of Columbia	25,576	66.0%	56.4%	32,933	85.0%	72.6%
Florida	446,201	44.2%	41.4%	799,782	79.2%	74.2%
Georgia	221,440	35.1%	31.7%	458,281	72.7%	65.6%
Hawaii	41,193	81.5%	64.4%	47,236	93.5%	73.8%
Idaho	68,736	81.0%	72.5%	78,461	92.5%	82.7%
Illinois	304,520	62.1%	49.3%	424,050	86.4%	68.7%
Indiana	273,987	85.7%	75.8%	301,762	94.4%	83.5%
Iowa	75,470	51.3%	46.7%	128,543	87.5%	79.5%
Kansas	105,576	83.7%	77.0%	115,630	91.6%	84.3%
Kentucky	177,470	70.0%	70.2%	219,533	86.6%	86.8%
Louisiana	276,097	84.8%	76.2%	306,093	94.0%	84.5%
Maine	38,574	81.1%	78.9%	42,781	89.9%	87.5%
Maryland	210,144	70.5%	72.2%	250,380	84.0%	86.0%
Massachusetts	149,308	67.6%	60.0%	189,252	85.7%	76.0%
Michigan	364,074	83.7%	71.5%	413,639	95.1%	81.3%
Minnesota	163,019	75.1%	71.1%	189,193	87.2%	82.6%
Mississippi	156,926	90.0%	67.8%	166,531	95.5%	72.0%
Missouri	20,384	8.3%	6.7%	150,002	61.1%	49.6%
Montana	23,006	54.2%	50.7%	35,874	84.6%	79.1%
Nebraska	57,393	71.4%	61.9%	67,726	84.3%	73.0%
Nevada	101,632	81.9%	65.5%	114,172	92.0%	73.6%
New Hampshire	23,330	79.4%	62.4%	25,132	85.6%	67.3%
New Jersey	-	0.0%	0.0%	133,337	43.7%	35.1%
New Mexico	125,568	86.0%	84.6%	135,724	93.0%	91.4%
New York	779,384	81.3%	72.3%	891,878	93.0%	82.7%
North Carolina	12,188	2.2%	2.1%	402,106	71.6%	69.2%
North Dakota	17,633	68.9%	67.1%	22,978	89.8%	87.5%
Ohio	438,582	77.1%	76.9%	507,439	89.2%	89.0%
Oklahoma	201,835	77.5%	74.1%	240,235	92.3%	88.2%
Oregon	118,314	64.2%	60.2%	168,213	91.2%	85.6%
Pennsylvania	412,217	86.4%	78.2%	438,983	92.1%	83.2%
Rhode Island	15,647	39.2%	34.7%	21,895	54.9%	48.6%
South Carolina	27,394	10.0%	9.4%	200,955	73.3%	69.1%
South Dakota	31,792	81.8%	72.1%	36,328	93.5%	82.4%
Tennessee	165,438	48.3%	39.8%	240,866	70.3%	58.0%
Texas	32,540	1.7%	1.6%	946,404	49.8%	47.9%
Utah	111,983	87.5%	79.1%	121,705	95.1%	85.9%
Vermont	16,633	69.8%	72.2%	18,562	77.9%	80.5%
Virginia	220,028	71.3%	68.7%	249,528	80.9%	77.9%
Washington	20,411	6.3%	5.5%	197,570	60.9%	53.6%
West Virginia	60,108	72.8%	56.4%	68,672	83.2%	64.5%
Wisconsin	196,524	84.8%	74.7%	216,201	93.3%	82.1%
Wyoming	20,491	81.4%	80.2%	22,993	91.3%	90.0%

References

American Medical Association. various. *Current Procedural Terminology: Professional Edition*. American Medical Association.

Centers for Disease Control and Prevention. n.d. "National Vital Statistics System - Restricted Data Access." https://www.cdc.gov/nchs/nvss/nvss-restricted-data.htm.

National Bureau of Economic Research. n.d. "National Plan and Provider Enumeration System (NPPES)." https://www.nber.org/research/data/national-plan-and-provider-enumeration-system-nppes.
